# Maternal Mediterranean diet in pregnancy and newborn DNA methylation: a meta-analysis in the PACE Consortium

**DOI:** 10.1080/15592294.2022.2038412

**Published:** 2022-03-02

**Authors:** Leanne K. Küpers, Sílvia Fernández-Barrés, Aayah Nounu, Chloe Friedman, Ruby Fore, Giulia Mancano, Dana Dabelea, Sheryl L. Rifas-Shiman, Rosa H. Mulder, Emily Oken, Laura Johnson, Mariona Bustamante, Vincent W.V. Jaddoe, Marie-France Hivert, Anne P. Starling, Jeanne H.M. de Vries, Gemma C. Sharp, Martine Vrijheid, Janine F. Felix

**Affiliations:** a The Generation R Study Group, Erasmus MC, University Medical Center Rotterdam, Rotterdam, The Netherlands; b Department of Pediatrics, Erasmus MC, University Medical Center Rotterdam, Rotterdam, The Netherlands; c ISGlobal, Institute for Global Health, Barcelona, Spain; d Universitat Pompeu Fabra (UPF), Barcelona, Spain; e Ciber Epidemiología Y Salud Pública (Ciberesp), Spain; f MRC Integrative Epidemiology Unit, University of Bristol, Bristol, UK; g Bristol Medical School Population Health Sciences, University of Bristol, Bristol, UK; h Department of Epidemiology, Colorado School of Public Health, University of Colorado Anschutz Medical Campus, Aurora, Colorado, USA; i Lifecourse Epidemiology of Adiposity and Diabetes (Lead) Center, University of Colorado Anschutz Medical Campus, Aurora, CO, USA; j Department of Population Medicine, Harvard Medical School, Harvard Pilgrim Health Care Institute, Boston, MA, USA; k Department of Pediatrics, School of Medicine, University of Colorado Anschutz Medical Campus, Aurora, CO, USA; l Centre for Exercise, Nutrition and Health Sciences, University of Bristol, Bristol, UK; m Diabetes Unit, Massachusetts General Hospital, Boston, MA, USA; n Department of Epidemiology, Gillings School of Global Public Health, University of North Carolina at Chapel Hill, Chapel Hill, NC, USA; o Division of Human Nutrition and Health, Wageningen University, Wageningen, The Netherlands

**Keywords:** Epigenetics, epigenome-wide association study, DNA methylation, Mediterranean diet, prenatal diet, maternal diet, newborn, cord blood

## Abstract

Higher adherence to the Mediterranean diet during pregnancy is related to a lower risk of preterm birth and to better offspring cardiometabolic health. DNA methylation may be an underlying biological mechanism. We evaluated whether maternal adherence to the Mediterranean diet was associated with offspring cord blood DNA methylation.

We meta-analysed epigenome-wide association studies (EWAS) of maternal adherence to the Mediterranean diet during pregnancy and offspring cord blood DNA methylation in 2802 mother–child pairs from five cohorts. We calculated the relative Mediterranean diet (rMED) score with range 0–18 and an adjusted rMED excluding alcohol (rMEDp, range 0–16). DNA methylation was measured using Illumina 450K arrays. We used robust linear regression modelling adjusted for child sex, maternal education, age, smoking, body mass index, energy intake, batch, and cell types. We performed several functional analyses and examined the persistence of differential DNA methylation into childhood (4.5–7.8 y).

rMEDp was associated with cord blood DNA methylation at cg23757341 (0.064% increase in DNA methylation per 1-point increase in the rMEDp score, SE = 0.011, *P* = 2.41 × 10^−8^). This cytosine–phosphate–guanine (CpG) site maps to *WNT5B*, associated with adipogenesis and glycaemic phenotypes. We did not identify associations with childhood gene expression, nor did we find enriched biological pathways. The association did not persist into childhood.

In this meta-analysis, maternal adherence to the Mediterranean diet (excluding alcohol) during pregnancy was associated with cord blood DNA methylation level at cg23757341. Potential mediation of DNA methylation in associations with offspring health requires further study.

## INTRODUCTION

The Mediterranean diet is a well-known dietary pattern with higher adherence related to decreased risk of cardiovascular disease and mortality [1,2]. In general, the Mediterranean diet is characterized by high consumption of vegetables, legumes, fruit, nuts, fish, cereals, monounsaturated vs. saturated fats (olive oil), and low-to-moderate consumption of alcohol, meat, and dairy products [2–4].

A healthy maternal diet during pregnancy is beneficial for maternal and offspring health. It has been associated with a reduced risk of maternal gestational diabetes [5], maternal hypertension [5] and better offspring cardiometabolic health [6]. Emerging evidence also suggests that maternal adherence to the Mediterranean diet during pregnancy is associated with lower risk of preterm birth, birth defects, and better offspring cardiometabolic and atopic health [7,8]. In pregnancy, the Mediterranean diet is defined slightly differently as compared to the original definition for the general public, because the alcohol component is considered detrimental in pregnancy and therefore mostly not included, and the dairy component is sometimes seen as beneficial, whereas that is not the case in the non-pregnant population [9].

Biological mechanisms underlying associations of maternal diet with offspring health are not yet completely understood, but one hypothesized mechanism is a mechanism via changes in offspring DNA methylation. Several epigenome-wide association study (EWAS) meta-analyses have tested associations of maternal exposures during pregnancy with cord blood DNA methylation [^10–14^], with some specifically focused on maternal nutritional status (e.g., body mass index (BMI), folate blood concentrations, or fatty acid supplementation) during pregnancy [13,15,16]. Two studies have explored maternal adherence to the Mediterranean diet in pregnancy and offspring DNA methylation, but these had small sample sizes (largest n = 390) and measured DNA methylation of CpGs in candidate regions [17,18]. Therefore, to improve statistical power and identify novel cord blood DNA methylation sites associated with maternal adherence to the Mediterranean diet during pregnancy, we performed a meta-analysis of epigenome-wide association studies (EWAS) in 2802 mother–child pairs from five cohorts in the Pregnancy And Childhood Epigenetics (PACE) Consortium [19].

## MATERIALS & METHODS

### Participants

Five cohorts participated in this meta-analysis: the Avon Longitudinal Study of Parents and Children (ALSPAC) from the UK, the Generation R Study from the Netherlands, the INfancia y Medio Ambiente (INMA) Project from Spain, and Healthy Start (two sub cohorts: Hispanic and non-Hispanic white participants) and Project Viva from the US. We excluded twins, and in case of non-twin siblings, we selected only one child per mother, based on completeness of data and, if equal, randomly. Each cohort performed complete case analyses; thus, mother–child pairs with missing data on maternal Mediterranean diet score, DNA methylation data or one or more of the covariates were excluded from analyses. Further explanation of cohort-specific analyses can be found in Supplementary File. Informed consent was obtained for all participants, procedures were in accordance with ethical standards of institution or regional committees and all cohorts had approval from their ethics boards (see cohort-specific methods in the Supplementary File).

### Maternal adherence to the Mediterranean diet during pregnancy

All cohorts obtained maternal dietary data through food frequency questionnaires (FFQs, ranging from 47 to 293 food items), except for Healthy Start, which used the Automated Self-Administered 24-hour dietary Assessment Tool [20] (ASA24) with a mean of 2.9 recalls (Supplementary Table 1).

All cohorts defined maternal adherence to the Mediterranean diet during pregnancy using the relative Mediterranean diet (rMED) score [4], based on a pre-specified common analysis plan and R script. The rMED is based on dietary data of consumption of the nine key food groups in Table 1. Cohort-specific deviations from this description are described in Supplementary File. For each food group, we calculated cohort-specific tertiles of intake and a score of 0, 1, or 2 was assigned to the first, second, or third tertile of intake of that specific food group, respectively. In the rMED, meat and dairy are given reversed scores; thus, higher consumption of meat and dairy lowered the total score. As per the original rMED definition, olive oil was scored as 0 for non-consumers, 1 for those below the median intake (in olive oil consumers) and 2 for those above the median intake in the individual cohort. We used a fixed dichotomous coding for alcohol based on the original definition where moderate alcohol consumption (5–25 g/d for women) fits within the Mediterranean diet (2 points) and lower or higher consumption was scored 0. We calculated the total rMED score by summing the scores for all food groups, resulting in a total score ranging between 0 and 18, with 18 representing the highest adherence to the Mediterranean diet. We additionally calculated an adjusted rMED excluding alcohol (rMEDp) from the score, as previously reported [21], because it is recommended not to consume alcohol during pregnancy. Since we aimed to stay as close as possible to the original Mediterranean diet definition and the evidence for benefits of dairy consumption during pregnancy is inconsistent, also for the pregnancy adjusted rMED, we kept the reversed scoring for the dairy component as in the original rMED. The total rMEDp therefore ranged between 0 and 16.

**Table 1.** Definition of food groups included in the relative Mediterranean diet (rMED) score with corresponding scoring per tertile.

Food group	rMED (Buckland et al. 2009)	rMEDp
	(g/100 0kcal/d)	(g/1000 kcal/d)
**1. Vegetables excl. Potatoes** **2. Legumes** **3. Fruit incl. nuts and seeds** **4. Fish and seafood** **5. Cereals**	Tertile 1 = 0 points	Tertile 1 = 0 points
	Tertile 2 = 1 point	Tertile 2 = 1 point
	Tertile 3 = 2 points	Tertile 3 = 2 points
**1. Meat** **2. Dairy products**	Tertile 1 = 2 points	Tertile 1 = 2 points
	Tertile 2 = 1 point	Tertile 2 = 1 point
	Tertile 3 = 0 points	Tertile 3 = 0 points
**Olive oil**	No consumption = 0 points	No consumption = 0 points
	<Median = 1 point	< Median = 1 point
	≥Median = 2 points	≥ Median = 2 points
**Alcohol in grams of ethanol/d (not energy density)**	5–25 g/d = 2 points	-
	Above or below 5–25 g/d = 0 points	
**Score range**	0–18 points	0–16 points

Meat and dairy products are reversely scored; thus, higher intake results in lower score.

In some instances, a food group in a specific cohort had a skewed distribution, making it impossible to calculate tertiles. In Generation R, for example, >33% of mothers reported to never consume legumes. Similarly, we saw skewed distributions for olive oil in ALSPAC, Healthy Start and Project Viva, with a large portion of the cohort never consuming it. Further, in Healthy Start legumes and fish also had skewed distributions, and in Project Viva this was also the case for legumes. In these situations, we set the first category to zero, where this first category includes >33% of the participants. We then defined the second and third categories by splitting the rest of the participants based on the median intake for those mothers who consumed the food item in question (see Supplementary File for all cohort-specific methods).

### Offspring cord blood DNA methylation

We measured offspring cord blood DNA methylation using the Illumina 450K array [22] and analysed this as untransformed DNA methylation beta-values ranging from 0 (completely unmethylated) to 1 (completely methylated). Cohorts performed their preferred laboratory analyses, quality control, and normalization of the DNA methylation data (Supplementary File). Each cohort excluded extreme DNA methylation beta-value outliers using the Tukey method [23]; outliers outside the range of (25th percentile – 3 × interquartile range (IQR)) to (75th percentile + 3 × IQR) were excluded.

### Statistical analyses

We used robust linear regression models for all analyses of the association of maternal adherence to the Mediterranean diet and each CpG site individually. Each cohort ran these robust linear regression models, using lmFit() using the Limma R package [24], following a pre-specified common analysis plan and R script. Each cohort ran four models, with the fully adjusted models (2 and 4) being the main models:

1. Newborn DNA methylation ~ rMED + sex + batch + cell types
2. Newborn DNA methylation ~ rMED + sex + maternal educational level + maternal age + maternal smoking + maternal BMI + maternal total energy intake + batch + cell types
3. Newborn DNA methylation ~ rMEDp (excluding the alcohol component) + sex + batch + cell types
4. Newborn DNA methylation ~ rMEDp (excluding the alcohol component) + sex + maternal educational level + maternal age + maternal smoking + maternal BMI + maternal total energy intake + batch + cell types

We defined maternal educational level in two or three levels, as per cohort definitions. Most cohorts classified maternal smoking into three groups: (1) No smoking in pregnancy, (2) Smoking, but stopped before second trimester, (3) Smoking throughout pregnancy, but two categories were used by ALSPAC (sustained smoking vs. no smoking or quit before second trimester) and Project Viva (any smoking vs. no smoking). We used maternal pre-pregnancy BMI, or early-pregnancy (<16 weeks’ gestation) BMI if pre-pregnancy BMI was not available in the cohort, in kg/m^2^. Maternal total energy intake (kcal/d) was calculated from the same dietary intake assessment as for Mediterranean diet score, with cohort-specific food composition tables. We estimated cell type composition (CD8T, CD4T, NK, B cells, monocytes, granulocytes, nucleated red blood cells) using the ‘Gervin’ reference set for cord blood DNA methylation [25]. Cohorts used their preferred variable to adjust for batch, e.g., plate, array number, or using surrogate variable analysis or ComBat [26]. We compared descriptives of all cohorts, including mean (standard deviation (SD)) and tertile cut-offs for both Mediterranean diet scores, and mean (SD) and tertile cut-offs for the food groups underlying these scores.

After running a quality control on all cohort results using the QCEWAS R package [27], fixed-effects inverse variance weighted meta-analyses of all cohort-specific results were performed using METAL [28] centrally at Erasmus MC. Shadow meta-analyses were performed at ISGlobal using GWAMA [29] and results were confirmed. Removing control probes (*N* = 65), probes mapping to the X chromosome (*N* = 11,231), or to the Y chromosome (*N* = 410) and those probes that cross-hybridized to alternate sequences, i.e., cross-reactive sites [30,31] (N = 44,960) resulted in a total of 429,701 CpGs to be tested in this meta-analysis. We flagged CpGs listed as potentially influenced by a single nucleotide polymorphism (polymorphic sites) by Naeem *et al.* [30] and Chen *et al*. [31] and those listed as methylation quantitative trait loci (mQTLs) [32]. We used a Bonferroni-corrected *P*-value threshold of *P* < 1.16 × 10^−7^ for statistical significance and we decided *a priori* to also present significant findings using a false discovery rate (FDR) *P*-value of <0.05 [33]. We performed all analyses in R, unless otherwise specified.

### Sensitivity analyses

To examine the robustness of the meta-analysis results, we performed a number of sensitivity analyses. We re-ran the meta-analysis in cohorts with participants of European ancestry only (ALSPAC, Generation R, Healthy Start non-Hispanic white, INMA, and Project Viva; the largest ancestry subgroup). Additionally, because Healthy Start used servings/1000 kcal/d and Project Viva used servings/d instead of g/1000 kcal/d as in the original rMED definition, and because Healthy Start did not use an FFQ, but the ASA24, we performed sensitivity analyses in European cohorts only (ALSPAC, Generation R, INMA) and in American cohorts only (Healthy Start Hispanic, Healthy Start NHW, Project Viva). We additionally performed leave-one-out analyses for the top CpG, to test the influence of single cohorts on the results.

### Look-up in childhood and adulthood

ALSPAC, Generation R, INMA, and Project Viva ran robust linear regression to assess the associations of maternal adherence to the Mediterranean diet during pregnancy and whole-blood DNA methylation measured in childhood (mean age ranging from 4.5 to 7.8 y). In these analyses, we looked up the CpG that survived Bonferroni correction in the main meta-analyses. These analyses included the same set of covariates, with the addition of child age at the time of DNA methylation sampling, and cell type composition (CD8T, CD4T, NK, B cells, monocytes, granulocytes) was estimated using the Houseman method with the ‘Reinius’ reference set [34,35]. We combined these results using a fixed-effects inverse variance weighted meta-analysis. We additionally did a lookup of the CpG, identified in the cord blood analyses, in a previously published cross-sectional meta-analysis that studied the association of Mediterranean diet with DNA methylation in adulthood [36] and looked up the 10 CpGs associated with Mediterranean diet in adulthood from that study in our cord blood analyses.

### Functional analyses

To explore functionality, we performed multiple analyses. First, we used the EWAS Catalog [37] to identify associations of the top CpG, and the gene it is mapped to, from the main meta-analysis with other traits previously published. Second, we checked whether the CpG associated with rMEDp also was associated with gene expression in childhood blood cells, measured as expression quantitative trait methylation (eQTM). For this analysis, we used the catalogue of 13.6 million blood autosomal cis-eQTMs in children by the Human Early Life Exposome (HELIX) project, after cell type adjustment [38]. Third, we ran functional enrichment analyses using Gene Ontology (GO) and Kyoto Encyclopedia of Genes and Genomes (KEGG) in MissMethyl [39] on tall CpGs with *P* < 0.0001 in the fully adjusted model for rMEDp. Fourth, we used eFORGE version 2.0 to examine enrichment for tissue-specific DNaseI hypersensitivity regions [40].

## RESULTS

### Participants

We included a total of 2802 mother–offspring pairs from five cohorts in this meta-analysis. We present cohort descriptive statistics in Table 2 and cohort-specific tertile cut-offs for the food groups that were used to construct the Mediterranean diet scores in Supplementary Table 2. Both the rMED and the rMEDp were normally distributed in all cohorts, with cohort means ranging from 6.1 to 8.7 and from 6.1 to 8.6, respectively (Table 2 and Supplementary Figure 1A-B).

**Table 2.** Cohort-specific descriptive statistics.

	ALSPAC	Generation R	Healthy Start^b^			
			Hispanic	Non-Hispanic white	INMA	Project Viva^b^
*N* total	660	1048	131	257	380	326
rMED	8.69 ± 2.83	7.79 ± 3.03	6.07 ± 2.15	6.89 ± 2.43	8.01 ± 2.62	7.88 ± 2.73
rMEDp excluding alcohol	8.57 ± 2.77	7.73 ± 3.00	6.07 ± 2.15	6.89 ± 2.43	7.98 ± 2.61	7.62 ± 2.66
Gender of the child – female	342 (51.8%)	523 (49.9%)	66 (50.4%)	134 (52.1%)	185 (48.7%)	161 (49.4%)
Maternal educational level^a^						
Low	320 (49.5%)	628 (59.9%)	77 (59.8%)	40 (15.6%)	101 (26.6%)	79 (24.2%)
Medium	-	-	-	-	162 (42.6%)	-
High	340 (51.5%)	420 (40.1%)	54 (41.2%)	217 (84.4%)	117 (30.8%)	247 (75.8%)
Maternal age (y)	29.8 ± 4.4	31.7 ± 4.1	24.5 ± 5.7	29.6 ± 5.1	31.6 ± 4.1	33.7 ± 4.5
Maternal smoking^a^						
No smoking during pregnancy	589 (89.2%)	801 (76.4%)	113 (86.3%)	216 (84.0%)	269 (70.8%)	429 (97.0%)
Smoked, stopped before 2nd trimester	-	103 (9.8%)	7 (5.3%)	19 (7.4%)	58 (15.3%)	29 (8.9%)
Smoked throughout pregnancy	71 (10.8%)	144 (13.7%)	11 (8.4%)	22 (8.6%)	53 (13.9%)	-
Maternal BMI (kg/m^2^)	22.8 ± 3.7	23.4 ± 3.9	28.6 ± 8.4	24.4 ± 5.4	23.8 ± 4.4	24.3 ± 9.4
Maternal total energy intake (kcal/d)	1754 ± 456	2156 ± 496	2000 ± 885	2076 ± 599	2069 ± 483	2119 ± 566
Vegetables						
g/d	135.8 ± 69.4	154.1 ± 62.5	-	-	243.4 ± 122.9	-
Servings/d	-	-	1.07 ± 0.66	1.25 ± 0.71	-	2.81 ± 1.54
Legumes						
g/d	53.9 ± 34.0	4.2 ± 6.1	-	-	30.8 ± 22.2	-
Servings/d	-	-	0.17 ± 0.30	0.11 ± 0.18	-	0.17 ± 0.30
Fruit and nuts						
g/d	110.8 ± 55.2	204.5 ± 113.1	-	-	263.1 ± 177.4	-
Servings/d	-	-	2.02 ± 2.14	2.64 ± 1.78	-	3.47 ± 1.69
Fish (g/d)^b^						
g/d	36.5 ± 29.4	13.2 ± 11.9	-	-	55.4 ± 32.1	-
Servings/d	-	-	0.65 ± 1.74	0.32 ± 0.75	-	0.23 ± 0.19
Cereals (g/d)^b^						
g/d	239.42 ± 120.70	207.2 ± 73.9	-	-	159.5 ± 56.5	-
Servings/d	-	-	6.56 ± 3.32	6.93 ± 2.52	-	4.08 ± 1.89
Meat (g/d)^b^						
g/d	70.1 ± 40.2	83.9 ± 39.1	-	-	128.9 ± 48.9	-
Servings/d	-	-	3.80 ± 2.90	3.03 ± 2.03	-	1.10 ± 0.65
Dairy (g/d)^b^						
g/d	391.7 ± 157.3	479.0 ± 247.6	-	-	411.2 ± 219.7	-
Servings/d	-	-	1.83 ± 1.25	2.34 ± 1.39	-	3.42 ± 1.58
Olive oil (g/d)^b^						
g/d	0.08 ± 0.08	5.5 ± 5.0	-	-	23.9 ± 12.3	-
Servings/d	-	-	0.00 ± 0.00	0.21 ± 0.90		0.11 ± 0.29
Alcohol (g/d)^b^	1.2 ± 3.0	0.9 ± 2.0	0.01 ± 0.07	0.10 ± 0.46	0.3 ± 1.4	2.43 ± 2.89
Alcohol intake of 5–25 g/d	39 (5.9%)	32 (3.1%)	0 (0%)	0 (0%)	6 (1.6%)	43 (13.2%)

Results presented as mean ± SD or *N* (%)

^a^Cohorts used their preferred categories for maternal educational level and maternal smoking during pregnancy. Please see the cohort-specific methods for these descriptions.

^b^Healthy Start used servings/1000 kcal/d and Project Viva used servings/d instead of g/1000 kcal/d as in the original rMED definition and as was used in the other cohorts.

### Meta-analysis

**Figure 1.**
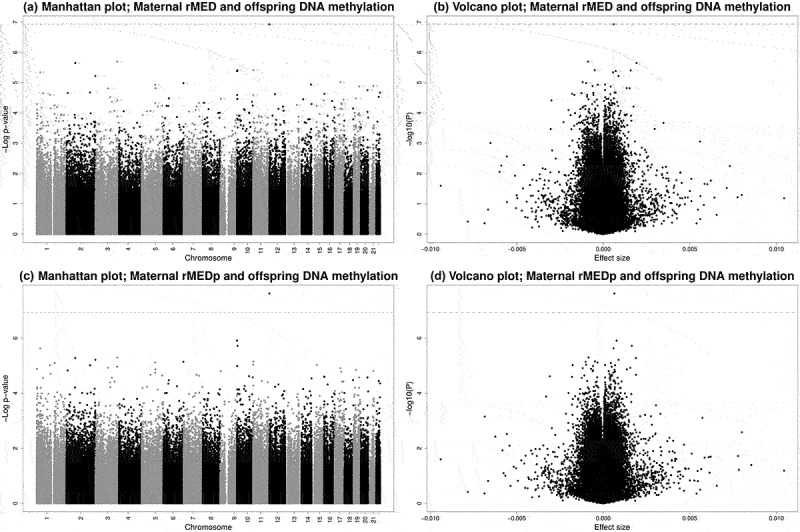
Manhattan and volcano plots of EWAS results for associations between maternal adherence to the Mediterranean diet, both including (rMED, a and b) and excluding the alcohol component (rMEDp, c and d) and offspring DNA methylation. Dashed lines represent the Bonferroni-corrected p-value threshold of *P* < 1.16 × 10^−7^.

We present full results from the meta-analyses in Supplementary Tables 3–6 and Supplementary Figure 2A-B, with CpGs with *P* < 1 × 10^−5^ from the main models in Tables 3 and 4. Manhattan and Volcano plots are presented in Figure 1. After adjusting for all covariates, rMEDp (excluding the alcohol component) was associated with cord blood DNA methylation at cg23757341 (0.064% increase in DNA methylation per 1-point increase in the rMEDp score, SE = 0.011, *P* = 2.41 × 10^−8^, I^2^ = 50.6). While we did not detect individual CpGs at which cord blood DNA methylation was significantly associated with the original rMED, cg23757341 also had the smallest *P*-value for the rMED (β = 0.060% increase in DNA methylation per 1-point increase in the rMED score, SE = 0.011, *P* = 1.19 × 10^−7^, I^2^ = 48.8), though just above the pre-specified Bonferroni significance threshold.

**Table 3.** Epigenome-wide associations of maternal adherence to the Mediterranean diet during pregnancy (rMED) and offspring cord blood DNA methylation level for cytosine–phosphate–guanine sites (*P* < 1 × 10^−5^).

MarkerName	Effect^a^	SE^a^	*P* value	Direction^b^	I^2^	Chr	Position	Gene Region	Relation to Island	Nearest Gene	Polymorphic	mQTL	FDR
cg23757341	0.0601	0.0114	1.19E-07	++++++	49	12	1,725,036	TSS1500	OpenSea	*WNT5B*	No	No	0.0510
cg09738156	−0.0880	0.0185	2.02E-06	– –	0	3	183,543,587	TSS200	Island	*MAP6D1*	No	Yes	0.2789
cg20348703	0.1918	0.0405	2.25E-06	+??+++	0	2	74,619,566	Body	S_Shore	*DCTN1-AS1*	Yes	Yes	0.2789
cg26053358	−0.0491	0.0106	3.87E-06	– –	53	11	2,397,576	TSS1500	N_Shore	*CD81*	Yes	No	0.2789
cg11894854	0.1581	0.0343	3.94E-06	++++++	7.7	10	5,237,853	TSS1500	OpenSea	*AKR1C4*	No	Yes	0.2789
cg13477178	0.0719	0.0156	4.15E-06	+++-++	0	10	375,248	Body	S_Shelf	*DIP2C*	Yes	Yes	0.2789
cg11747820	0.0563	0.0123	4.54E-06	++++++	0	1	29,584,023	Body	N_Shore	*PTPRU*	Yes	No	0.2789
cg07203767	0.0878	0.0194	6.02E-06	++++++	30	2	242,973,721		OpenSea	*LINC01881*	No	No	0.3234
cg26664457	−0.1193	0.0269	9.60E-06	– –	22	17	47,394,423	Body	OpenSea	*ZNF652*	No	No	0.4114

^a^Effect size and SE are presented as percentage change in DNA methylation per 1-point increase in the rMEDp score.

^b^Cohorts are ordered as follows: ALSPAC, Generation R, Healthy Start Hispanic, Healthy Start non-Hispanic White, INMA, Project Viva.

EWAS was adjusted for sex + maternal educational level + maternal age + maternal smoking + maternal BMI + maternal total energy intake + batch + cell types.

**Table 4.** Epigenome-wide associations of maternal adherence to the Mediterranean diet during pregnancy (rMEDp excl. the alcohol component) and offspring cord blood DNA methylation level for cytosine–phosphate–guanine sites (*P* < 1 × 10^−5^).

MarkerName	Effect^a^	SE^a^	*P* value	Directionb	I^2^	Chr	Position	Gene Region	Relation to Island	Nearest Gene	Polymorphic	mQTL	FDR
cg23757341	0.0637	0.0114	2.41E-08	++++++	51	12	1,725,036	TSS1500	OpenSea	*WNT5B*	No	No	0.01
cg13477178	0.0766	0.0158	1.24E-06	+++-++	0	10	375,248	Body	S_Shelf	*DIP2C*	Yes	Yes	0.252
cg11894854	0.1648	0.0346	1.92E-06	++++++	8.9	10	5,237,853	TSS1500	OpenSea	*AKR1C4*	No	Yes	0.252
cg11747820	0.0596	0.0126	2.35E-06	-+++++	5.9	1	29,584,023	Body	N_Shore	*PTPRU*	Yes	No	0.252
cg09738156	−0.0854	0.0187	5.05E-06	– –	0	3	183,543,587	TSS200	Island	*MAP6D1*	No	Yes	0.273
cg20348703	0.1883	0.0413	5.28E-06	+??+++	0	2	74,619,566	Body	S_Shore	*DCTN1-AS1*	Yes	Yes	0.273
cg07203767	0.0886	0.0196	6.15E-06	++++++	23	2	242,973,721		OpenSea	*LINC01881*	No	No	0.273
cg26053358	−0.0481	0.0107	7.07E-06	– –	50	11	2,397,576	TSS1500	N_Shore	*CD81*	Yes	No	0.273
cg11529346	−0.1017	0.0227	7.27E-06	– –	0	6	168,665,455		OpenSea	*DACT2*	No	Yes	0.273
cg14773728	−0.0328	0.0073	7.73E-06	-++ –	33	5	112,312,254	TSS200	N_Shore	*DCP2*	No	No	0.273
cg11946165	0.0436	0.0098	8.02E-06	++++++	66	1	150,781,434	TSS1500	OpenSea	*CTSK*	No	No	0.273
cg20239381	−0.0992	0.0224	9.21E-06	– –	0	11	590,115	Body	Island	*PHRF1*	Yes	No	0.273
cg24007300	0.0104	0.0024	9.55E-06	+-+–+	0	15	42,264,860	TSS200	Island	*EHD4*	No	No	0.273
cg21217540	−0.1769	0.0400	9.59E-06	– –	13	1	110,752,307		Island	*KCNC4*	Yes	Yes	0.273
cg11994984	0.0682	0.0154	9.73E-06	++++-+	42	2	198,364,525	1stExon; 5ʹUTR; 5ʹUTR; TSS200	Island	*HSPD1*	No	Yes	0.273

^a^Effect size and SE are presented as percentage change in DNA methylation per 1-point increase in the rMEDp score.

^b^Cohorts are ordered as follows: ALSPAC, Generation R, Healthy Start Hispanic, Healthy Start non-Hispanic White, INMA, Project Viva.

EWAS was adjusted for sex + maternal educational level + maternal age + maternal smoking + maternal BMI + maternal total energy intake + batch + cell types.

### Sensitivity analyses

To assess the robustness of the finding, we performed several sensitivity analyses for the top CpG from the main meta-analysis in the fully adjusted models for rMEDp (Table 5). First, from the meta-analysis in European ancestry cohorts (thus excluding Healthy Start Hispanic) we obtained results very similar to the main meta-analysis. Sensitivity analyses without both sub-cohorts of the Healthy Start study, using ASA24 and not FFQ, resulted in a slightly lower effect size (0.049% change in DNA methylation per 1-point increase in the rMEDp compared to 0.064% in the main meta-analysis), lower heterogeneity I^2^, but higher *P*-value. When removing cohorts that defined dietary assessment in servings instead of grams, either Healthy Start (both sub cohorts), Project Viva, or both, the effect size decreased slightly (ranging between 0.038% and 0.055% change in DNA methylation per 1-point increase in the rMEDp) and *P*-values did not reach Bonferroni significance anymore. When meta-analysing only Healthy Start and Project Viva, the effect size increased to 0.094% change in DNA methylation per 1-point increase in the rMEDp (compared to 0.064% in the main meta-analysis) with a similar *P*-value (*P* = 2.31 × 10^−8^) as identified in the main meta-analysis and lower heterogeneity (I^2^ = 16.5). We show a forest plot and a leave-one-out plot for cg23757341 in Supplementary Figures 3 and 4, with the largest change in effect size observed when excluding Healthy Start non-Hispanic white population from the meta-analysis (−18.2%). To additionally test a potentially systematic pattern, we constructed forest plots for the top three CpGs associated with rMEDp and did not see similar heterogeneity in forest plots for cg13477178 and cg11894854 (Supplementary Figure 5).

**Table 5.** Sensitivity analyses for the top hit of the main meta-analysis (cg23757341).

	*N*	Effect^a^	SE^a^	*P* value	Direction	I^2^
*Main meta-analysis*						
rMEDp fully adjusted	2802	0.0638	0.0114	2.41E-08	++++++	50.6
rMED fully adjusted	2802	0.0601	0.0114	1.19E-07	++++++	48.8
*Main meta-analysis*						
rMEDp minimally adjusted	2802	0.0500	0.0109	4.73E-06	++++++	52.1
rMED minimally adjusted	2802	0.0467	0.0108	1.45E-05	++++++	51.5
*Sensitivity analyses for rMEDp fully adjusted*						
European ancestry only (ALSPAC, Generation R, Healthy Start NHW, INMA, Project Viva)	2671	0.0660	0.0119	2.67E-08	+++++	58.4
Cohorts using FFQ only (ALSPAC, Generation R, INMA, Project Viva)	2414	0.0491	0.0133	2.21E-04	++++	38.8
European cohorts only (ALSPAC, Generation R, INMA)	2088	0.0378	0.0156	1.51E-02	+++	0
American cohorts only (Healthy Start Hispanic, Healthy Start NHW, Project Viva)	714	0.0939	0.0168	2.31E-08	+++	16.5
*Leave-one-out analyses for rMEDp fully adjusted*						
Excluding ALSPAC	2142	0.0680	0.0120	7.90E-09	+++++	49.7
Excluding Generation R	1754	0.0682	0.0139	8.61E-07	+++++	59.2
Excluding Healthy Start Hispanic	2671	0.0660	0.0119	2.67E-08	+++++	58.4
Excluding Healthy Start Non Hispanic White	2545	0.0522	0.0128	4.43E-05	+++++	33.4
Excluding INMA	2422	0.0714	0.0124	7.56E-09	+++++	46.5
Excluding Project Viva	2476	0.0550	0.0127	1.25E-05	+++++	48.8

^a^Effect size and SE are presented as percentage change in DNA methylation per 1-point increase in the rMEDp score.

NHW, non-Hispanic white.

EWAS was adjusted for sex + maternal educational level + maternal age + maternal smoking + maternal BMI + maternal total energy intake + batch + cell types.

### Look-up in childhood and adulthood

We did not observe an association of maternal adherence to the Mediterranean diet during pregnancy with DNA methylation at cg23757341 in childhood blood (cohort descriptive statistics in Supplementary Table 7, *n* = 1593, β = −0.0022% change in DNA methylation per 1-point increase in the rMEDp score, SE = 0.016, *P* = 0.89, and for all individual cohorts *P* > 0.05, Table 6).

**Table 6.** Meta-analysis results for associations between maternal adherence to the Mediterranean diet during pregnancy and methylation level (cg23757341) in childhood (mean age 4.5–7.8 y).

	*N*	Effect^a^	SE^a^	*P* value	Direction^b^	I^2^
rMEDp fully adjusted	1593	−0.0022	0.0165	8.92E-01	+–+	38
rMED fully adjusted	1593	−0.0055	0.0163	7.35E-01	+–+	43

^a^Effect size and SE are presented as percentage change in DNA methylation per 1-point increase in the rMEDp score.

^b^Cohorts are ordered as follows: ALSPAC, Generation R, INMA, Project Viva.

EWAS was adjusted for sex + age of the child at DNA methylation sampling + maternal educational level + maternal age + maternal smoking + maternal BMI + maternal total energy intake + batch + cell types.

In a previous cross-sectional meta-analysis in adults, cg23757341 was not among the hits associated with adherence to own adherence Mediterranean diet [36]. Additionally, none of the 10 CpGs found to be associated with Mediterranean diet in adults reached significance in our cord blood EWAS for rMED and rMEDp (all *P* ≥ 0.04) and all were flagged as either an mQTL [32] or as potentially polymorphic [30,31] (Supplementary Table 8).

### Functional analyses

To examine potential functionality of the main hit we ran several analyses, after annotating the CpG and finding that it maps to the transcription start site of the *WNT5B* gene, known to be associated with adipogenesis, insulin secretion, and type 2 diabetes [41]. First, a lookup in the EWAS Catalog indicated that DNA methylation at cg23757341 in adult whole blood was previously found to be marginally associated with fasting insulin [42] (*P* = 7.8 × 10^−5^). A look-up on 30 June 2021 of the *WNT5B* gene in the EWAS Catalog showed 204 associations of 42 CpGs annotated to this gene with multiple phenotypes, mostly gestational and childhood age. Second, the lookup of cg23757341 in the catalogue of gene expression in blood autosomal cis-eQTMs in children [38] showed no significant CpG – transcript cluster associations after multiple testing correction (Supplementary Table 9). Third, functional enrichment analyses on the 88 CpGs with *P* < 0.0001 in the fully adjusted model for rMEDp did not result in FDR significant pathways for both GO and KEGG; results with *P* < 0.01 are presented in Supplementary Table 10 (none of the KEGG pathways reached *P* < 0.01 and therefore not presented in this table). Fourth, there was no evidence of enrichment for tissue-specific DNaseI hypersensitivity regions.

## DISCUSSION

In this meta-analysis of five population-based cohort studies, we found that maternal adherence to the Mediterranean diet during pregnancy was associated with cord blood DNA methylation at one CpG, cg23757341. This CpG maps to the transcription start site of the *WNT5B* gene. We did not identify persistence of the differential methylation into childhood blood for cg23757341.

A higher adherence to a Mediterranean diet of mothers during pregnancy has been related to lower risk of preterm birth, birth defects, and better offspring cardiometabolic and atopic health [7,8]. Differential DNA methylation may represent an underlying mechanism. Thus, we hypothesized that maternal adherence to the Mediterranean diet would be associated with DNA methylation in cord blood. The positive association of DNA methylation at cg23757341 with maternal adherence to the Mediterranean diet in the current analysis and the previously published (marginal) negative association of DNA methylation at cg23757341 with fasting insulin in adult whole blood [42] could support a potential mechanism underlying this association. Indeed previously, overexpression of *WNT5B* was associated with increased adipogenesis in adipocytes, suggesting a role in type 2 diabetes development [43]. Moreover, this CpG is located in the transcription start site of the *WNT5B* gene, known to be associated with adipogenesis, insulin secretion, and type 2 diabetes [41], which could represent a potential biological mechanism via DNA methylation. However, we did not identify enriched biological pathways for 88 CpGs, only one of which is the main hit, or a known link to childhood blood gene expression. Further research is needed to confirm a mediating mechanism to offspring later life health.

The only other epigenome-wide association study on adherence to the Mediterranean diet and DNA methylation was performed in adults [36] and their 10 hits did not show overlap with our findings. Previous studies have observed associations of maternal intake during pregnancy of individual nutrients (e.g., folate [13] and fatty acids [15]) with DNA methylation, which may help to elucidate biological mechanisms to offspring health outcomes. However, nutrients are not consumed in isolation but as part of a complex dietary pattern, therefore studies of more holistic dietary exposures, such as the Mediterranean diet that capture interactions between foods and nutrients consumed together, are arguably more relevant for a public health interpretation.

We explored several potential explanations for the marginally high heterogeneity I^2^ (50.6%) for cg23757341 and could not find any major methodological issues as the underlying cause for this. First, Healthy Start was the only cohort to use a 24 h dietary assessment method instead of an FFQ. However, based on the leave-one-out plot there seems to only be an effect of excluding the Healthy Start non-Hispanic white group and not the Healthy Start Hispanic group. Thus, the different dietary assessment methods do not seem to be the main cause of high heterogeneity. Second, both US-based cohorts had dietary data available in servings instead of grams. However, the rMED was constructed using cohort-specific tertile cut-offs and not absolute values. We used these cohort-specific tertiles, because the ranking of individuals will not be affected; therefore, this difference in units unlikely explains the heterogeneity. Both the European cohort-specific and the US cohort-specific meta-analyses showed no or low heterogeneity, indicating that there may be an unexplained difference between the European and US cohorts in this meta-analysis, geographical locations may be contributing to the overall heterogeneity. However, we did not observe a systematic pattern for the two CpGs with the second and third smallest *P*-values. Last, the variety and range of foods eaten has expanded worldwide over the last 30 y. This could have resulted in differences in dietary data between cohorts due to differences in time of data collection, i.e., early 1990s for ALSPAC and around 2010 for Healthy Start. We aimed to minimize a potential effect of this by standardizing the between-cohort variation using tertile cut-offs in the rMED.

This is the first large-scale meta-analysis of multiple epigenome-wide association studies to study the association between maternal adherence to the Mediterranean diet during pregnancy and offspring cord blood DNA methylation. As is common in the PACE consortium, all cohorts used a pre-defined uniform analysis plan for the calculation of rMED and for running the EWAS, limiting the between-cohort variation. Another strength of this study is the fact that we included mother–offspring pairs from multiple countries. Potential limitations of this study include the fact that self-reported questionnaires [44,45] were used in all cohorts, which could have introduced measurement error. Each cohort performed their own preferred quality control and normalization, which did not largely affect associations in previous studies in the PACE consortium [10]. Additionally, the rMED is based on cohort-specific relative food tertile cut-offs, which might have challenged true comparability across cohorts. However, a previous paper showed similar satisfactory assessment of adherence to the Mediterranean diet, independent of the Mediterranean diet score of choice [46]. Nevertheless, these results should be interpreted in the context of Western countries, that nowadays have lower consumption of the Mediterranean plant-based diet than traditionally in Mediterranean countries. For example, intake of vegetables was rather low in ALSPAC even in the highest tertile. If it is the case that an association only occurs above a certain threshold of intake, it might be, that this relatively low intake did not meet that threshold. Additionally, the time of collection of food frequency questionnaires varied between cohorts (Supplementary Table 1). However, we do not expect a substantial change of adherence to the Mediterranean diet throughout pregnancy, since Project Viva has previously assessed diet during pregnancy at two time points with stable dietary intake during pregnancy and high correlation of Mediterranean diet score when comparing the first and second trimester [47,48]. Another potential limitation might be that magnitudes of associations were small. However, small DNA methylation differences can have functionally relevant consequences [49]. As in all observational studies causality is difficult to study. A comparison between maternal and paternal exposure could potentially help establish causality using the paternal data as a negative control, but unfortunately paternal FFQs are less often available in observational cohort studies. In fact, we explored the possibility of generating rMED for fathers in ALSPAC, but the FFQs existed of too few food items in the dataset to generate rMED. Additionally, Mendelian randomisation might increase understanding of potential causality but instrumental variables for Mediterranean diet are not (yet) available.

To conclude, in this meta-analysis, maternal adherence to the Mediterranean diet during pregnancy was associated with cord blood DNA methylation level at one CpG located in the *WNT5B* gene. A potential mediating role of DNA methylation in the association of maternal diet and offspring health requires further study.

## Supplementary Material

Supplemental Material

## Data sharing statement

Data described in the manuscript, code book, and analytic code will be made available upon request pending application and approval. Full EWAS results can be found at doi:10.5281/zenodo.5877662. Total files are approximately 46MB.
