# Supplementary material for: Maternal Mediterranean diet in pregnancy and newborn DNA methylation: a meta-analysis in the PACE Consortium
Source: Epigenetics. 2022 Mar 2;17(11):1419–31. doi: 10.1080/15592294.2022.2038412 (PMC9586614; doi:10.1080/15592294.2022.2038412)
Supplement: Supplemental Material [file KEPI_A_2038412_SM6220.docx]

**Maternal Mediterranean diet in pregnancy and newborn DNA methylation: a meta-analysis in the PACE Consortium**

**Supplementary File**

**Contents**

[**Supplementary figures** 3](#_Toc78202695)

[**Supplementary Figure 1**. Cohort-specific distribution of the rMED (A) and the rMEDp (B). 3](#_Toc78202696)

[**Supplementary Figure 2**. QQ plots and lambdas for fully adjusted EWAS for the association of cord blood DNA methylation with A) maternal adherence to rMED and B) maternal adherence to rMEDp. 4](#_Toc78202697)

[**Supplementary Figure 3**. Forest plot for cg23757341. 4](#_Toc78202698)

[**Supplementary Figure 4**. Leave-one-out plot for cg23757341, for the influence of each individual cohort on the meta-analysis result, the vertical reference line is the main meta-analysis result effect size. 5](#_Toc78202699)

[**Supplementary Figure 5**. Forest plots for the two other CpGs in the top 3 with the smallest p-values for the association with rMEDp; A) cg13477178 and B) cg11894854. 6](#_Toc78202700)

[**Supplementary Tables** 7](#_Toc78202701)

[**Supplementary Table 1**. Description of food questionnaires used in each cohort 7](#_Toc78202702)

[**Supplementary Table 2**. Tertile cut-offs for each food component and the rMED score, as defined in each cohort. 8](#_Toc78202703)

[**Supplementary Table 3.** Full EWAS results of rMED and offspring cord blood DNA methylation – minimally adjusted (*publicly available online upon acceptance of the manuscript*). 10](#_Toc78202704)

[**Supplementary Table 4.** Full EWAS results of rMEDp and offspring cord blood DNA methylation – minimally adjusted (*publicly available online upon acceptance of the manuscript*). 10](#_Toc78202705)

[**Supplementary Table 5.** Full EWAS results of rMED and offspring cord blood DNA methylation – fully adjusted (*publicly available online upon acceptance of the manuscript*). 10](#_Toc78202706)

[**Supplementary Table 6.** Full EWAS results of rMEDp and offspring cord blood DNA methylation – fully adjusted (*publicly available online upon acceptance of the manuscript*). 10](#_Toc78202707)

[**Supplementary Table 7.** Cohort descriptives for the look up for association of maternal adherence to the Mediterranean diet during pregnancy with DNA methylation (cg23757341) measured in childhood. 11](#_Toc78202708)

[**Supplementary Table 8.** Lookup of CpGs previously associated with Mediterranean diet in adults (Ma J et al. Circ Genom Precis Med 2020). 13](#_Toc78202709)

[**Supplementary Table 9**. Lookup of cg23757341 in the blood autosomal cis-eQTM catalogue from the Human Early Life Exposome (HELIX) project. 14](#_Toc78202710)

[**Supplementary Table 10.** Gene ontology (GO) results (P<0.01), based on n=81 CpGs with P<0.0001. 16](#_Toc78202711)

[**Supplementary cohort-specific methods** 18](#_Toc78202712)

[*The Avon Longitudinal Study of Parents and Children (ALSPAC)* 18](#_Toc78202713)

[*The Generation R Study* 21](#_Toc78202714)

[*Healthy Start* 24](#_Toc78202715)

[*The INMA—INfancia y Medio Ambiente—(Environment and Childhood) Project* 27](#_Toc78202716)

[*Project Viva* 29](#_Toc78202717)

[**References** 32](#_Toc78202718)

# Supplementary figures

**A)**


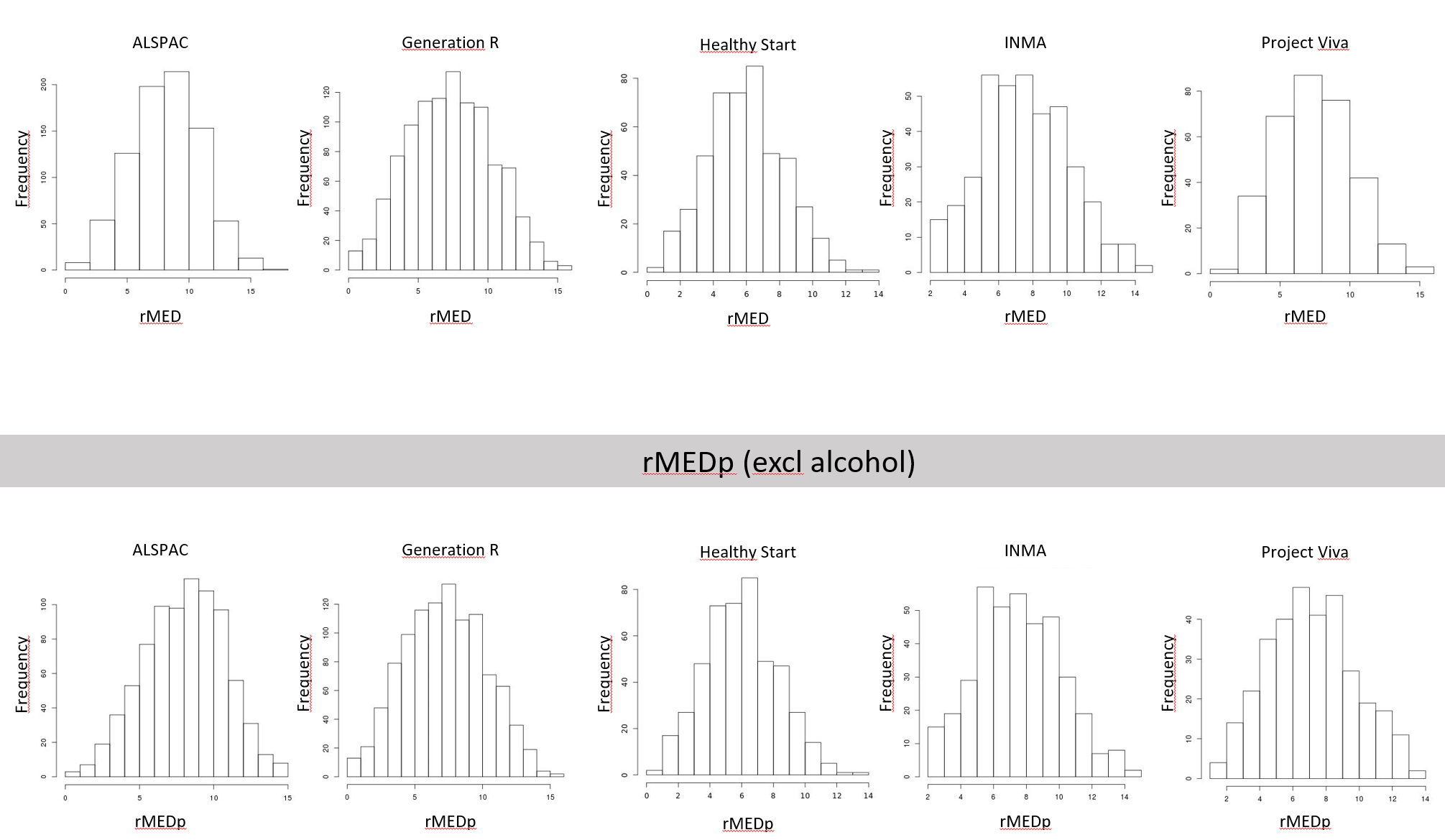


**B)**


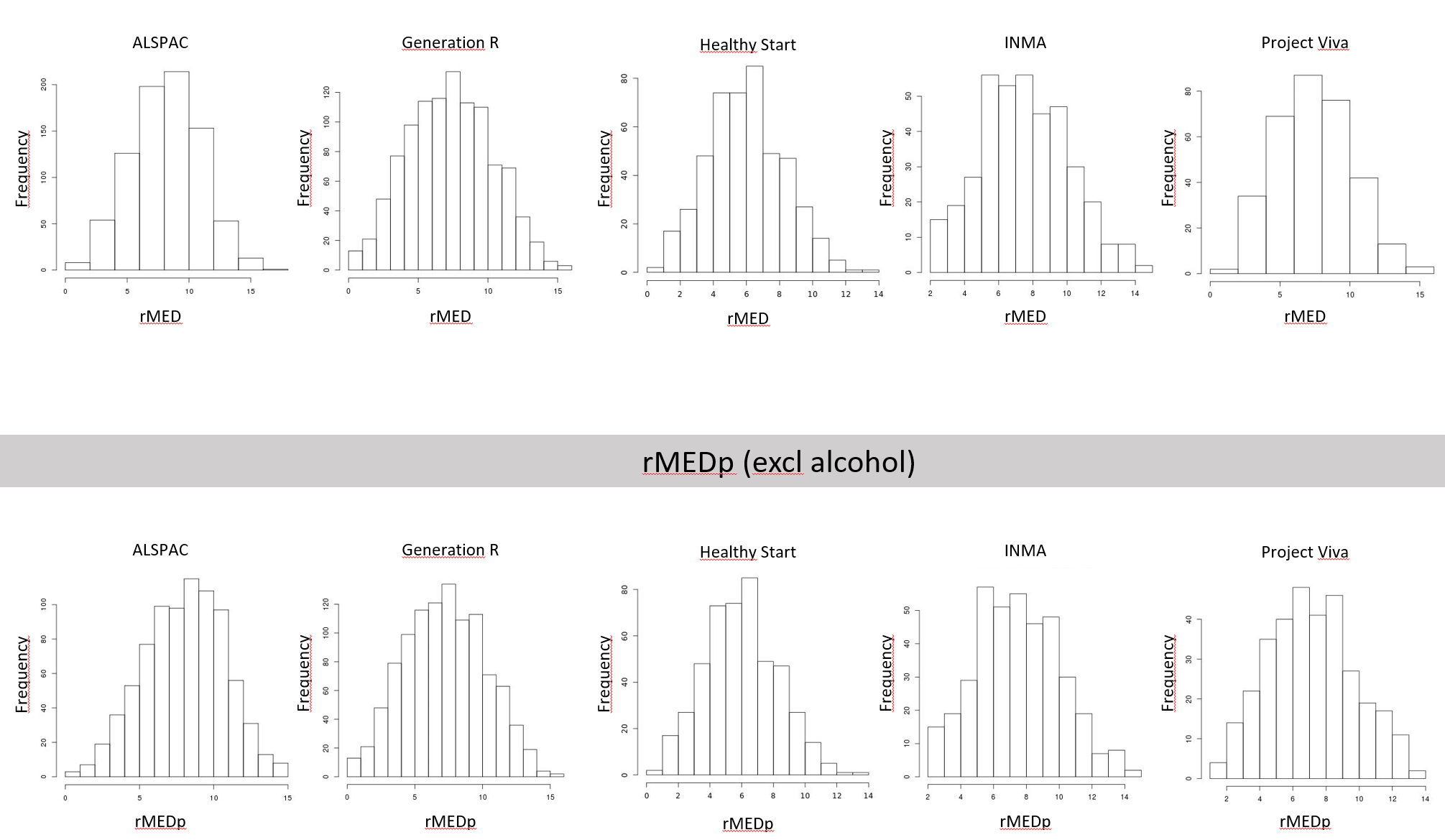


## **Supplementary Figure 1**. Cohort-specific distribution of the rMED (A) and the rMEDp (B).


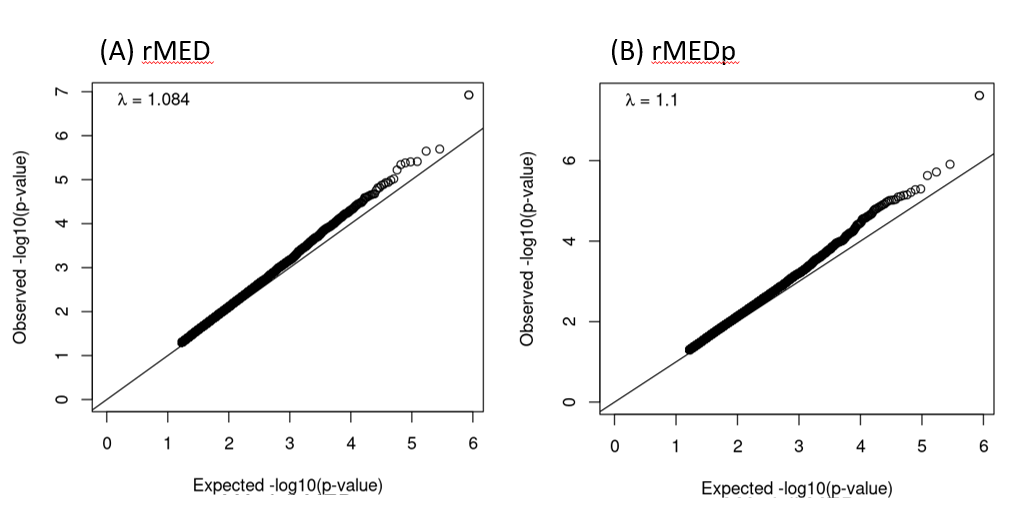


## **Supplementary Figure 2**. QQ plots and lambdas for fully adjusted EWAS for the association of cord blood DNA methylation with A) maternal adherence to rMED and B) maternal adherence to rMEDp.


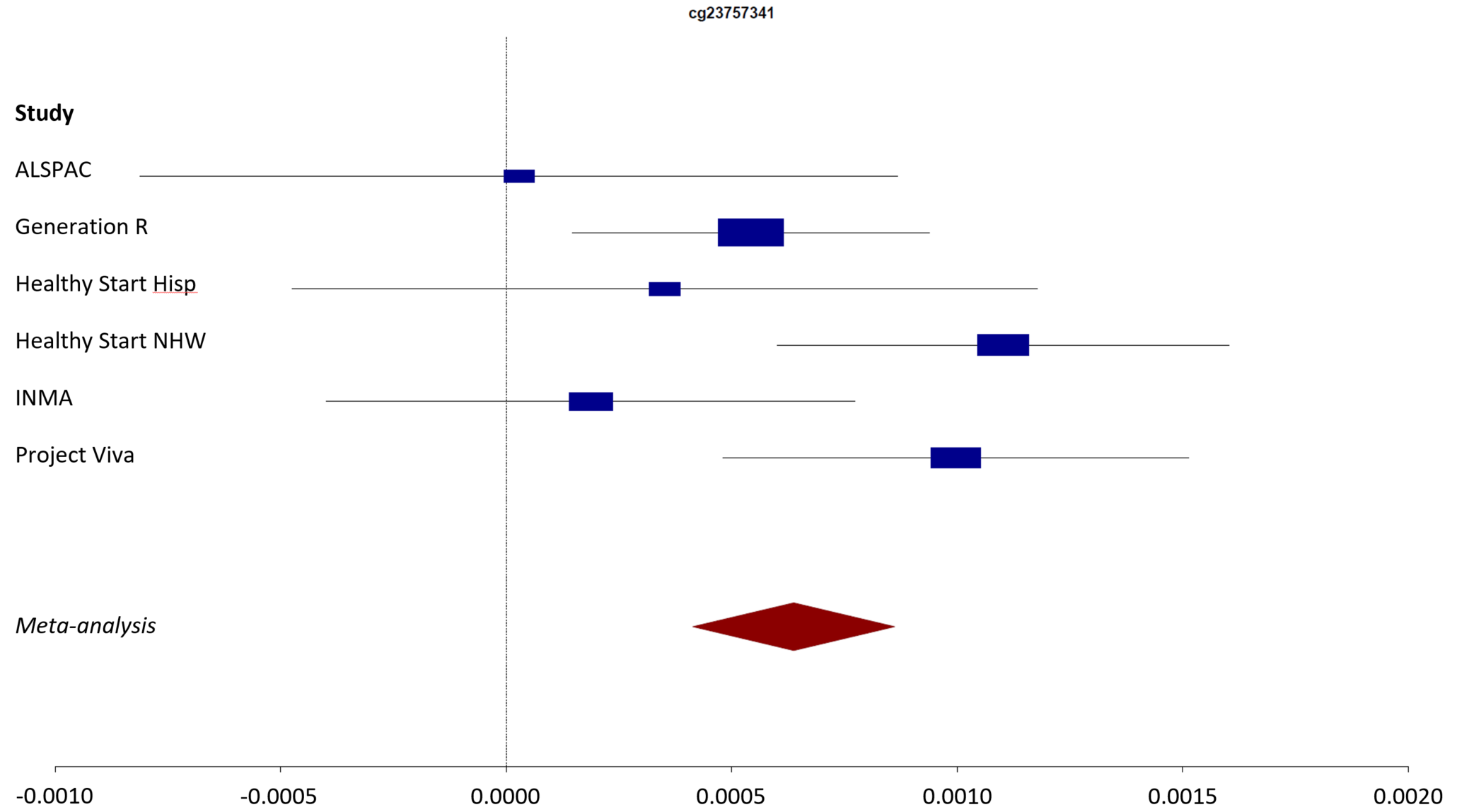


## **Supplementary Figure 3**. Forest plot for cg23757341.


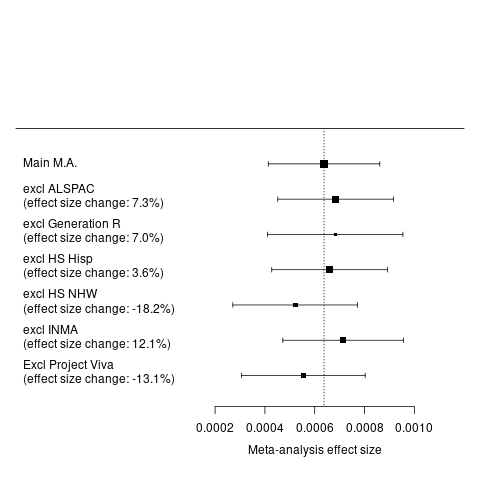


## **Supplementary Figure 4**. Leave-one-out plot for cg23757341, for the influence of each individual cohort on the meta-analysis result, the vertical reference line is the main meta-analysis result effect size.


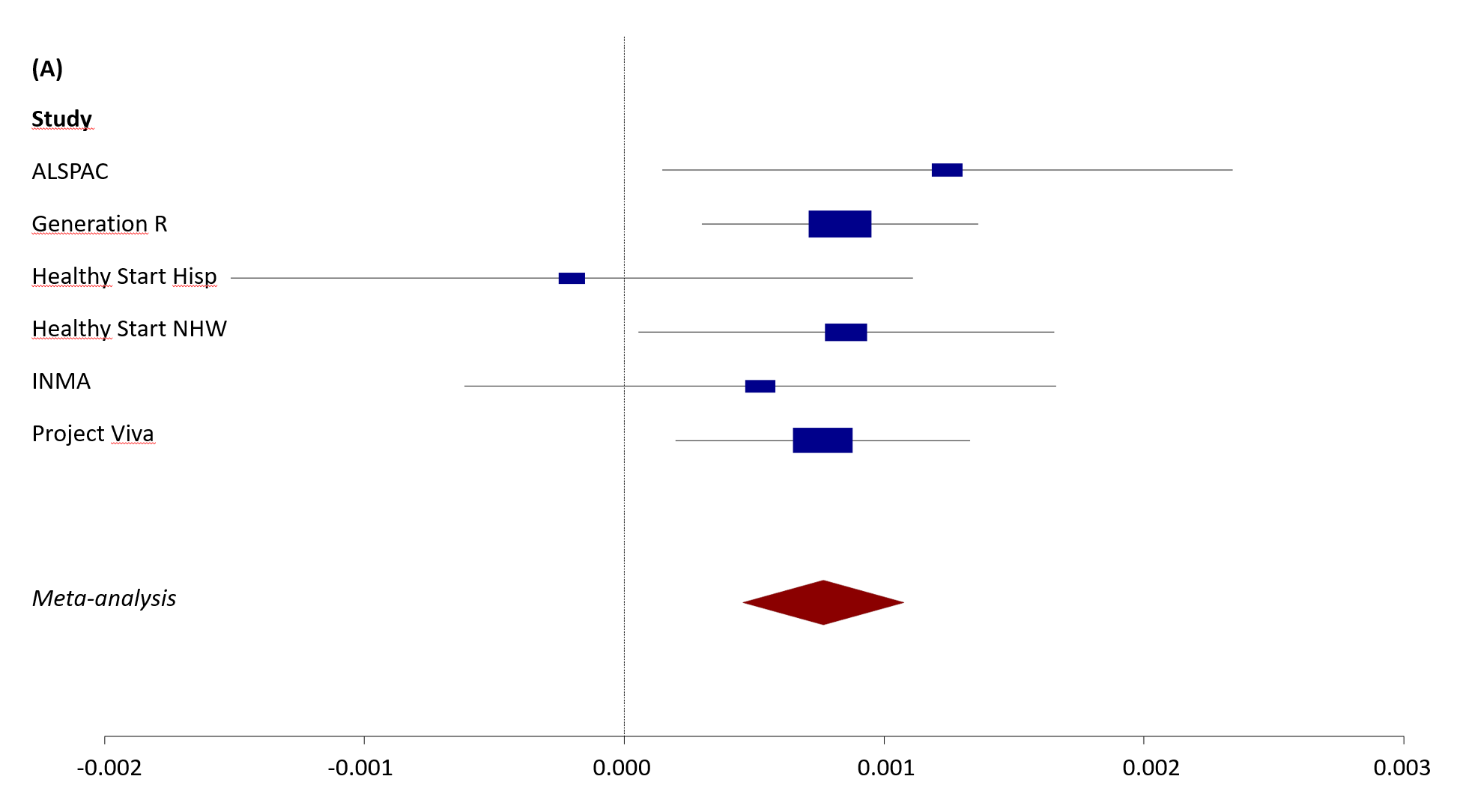

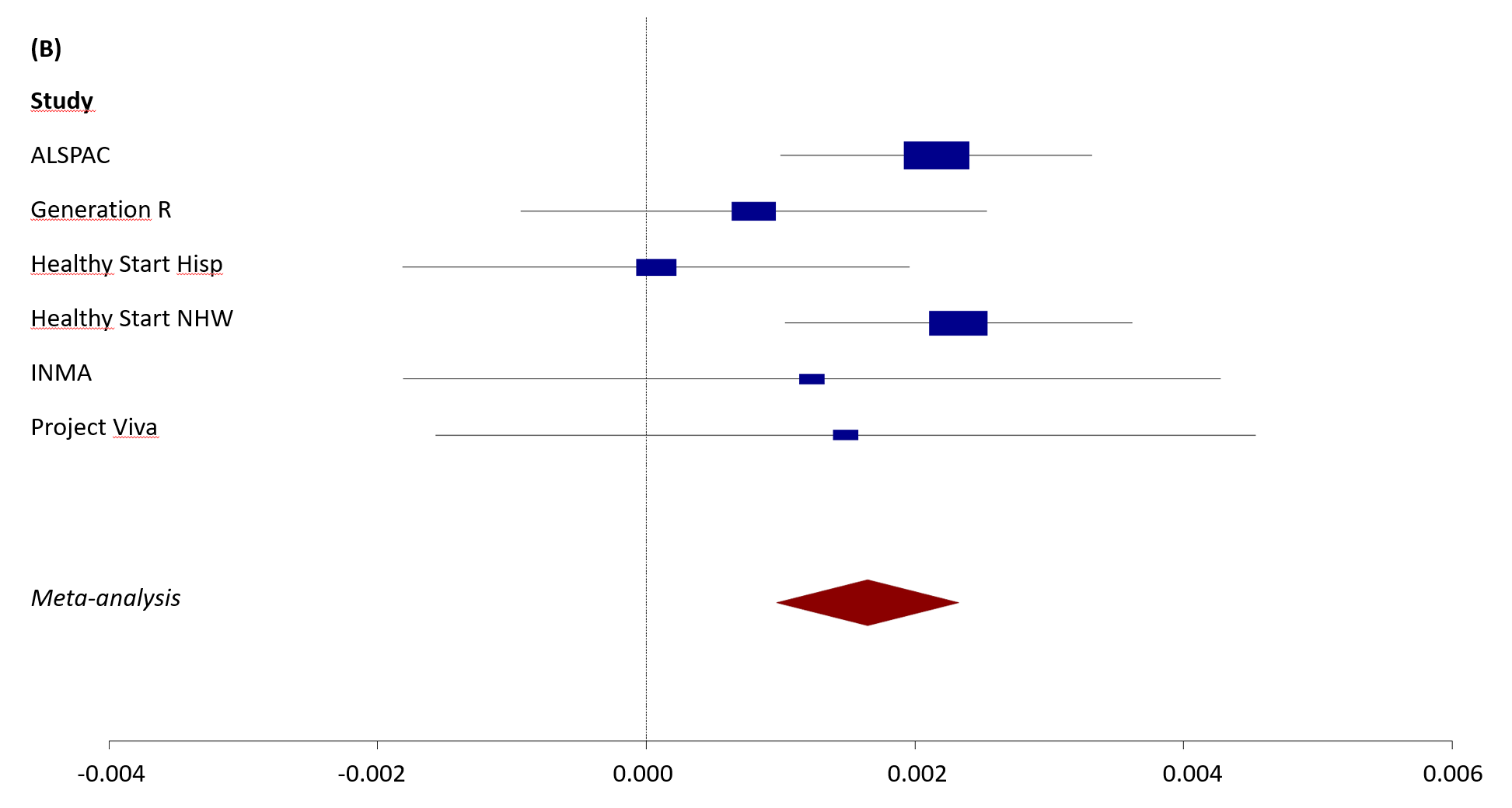


## **Supplementary Figure 5**. Forest plots for the two other CpGs in the top 3 with the smallest p-values for the association with rMEDp; A) cg13477178 and B) cg11894854.

# Supplementary Tables

## **Supplementary Table 1**. Description of food questionnaires used in each cohort

| **Study name** | **Food questionnaire** | **Trimester of food questionnaire** | **Reference for validation method** |
| --- | --- | --- | --- |
| ALSPAC | 47-item FFQ | 3^rd^ trimester | Emmett PM *et al*. Nutr Rev. 2015 |
| Generation R | 293-item FFQ | Median 13.5 weeks (95% range 10.1-21.8 weeks) | Klipstein-Grobusch K et al. Eur J Clin Nutr 1998 |
| Healthy Start | Automated Self-Administered 24-hour dietary Assessment Tool with a median of 2 recalls (range 1-8). | Median 25 weeks (range 9.7-39.9 weeks) | Moshfegh, AJ *et al*. Am J Clin Nutr. 2008 |
| INMA | 101-item FFQ | 1^st^ trimester | Vioque J et al. Nutr J. 2013 |
| Project Viva | 166-item semi quantitative FFQ | 1^st^ trimester | Fawzi WW et al. Annals of Epidemiol. 2004 |
| FFQ: food-frequency questionnaire | |  |  |

## **Supplementary Table 2**. Tertile cut-offs for each food component and the rMED score, as defined in each cohort.

|  | **ALSPAC** | |  | **Generation R** | |  | **Healthy Start Hisp** | |  | **Healthy Start NHW** | |  | **INMA** | |  | **Project Viva** | |  |
| --- | --- | --- | --- | --- | --- | --- | --- | --- | --- | --- | --- | --- | --- | --- | --- | --- | --- | --- |
|  | grams/1000kcal/day | |  | grams/1000kcal/day | |  | servings/1000kcal/day | |  | servings/100kcal/day | |  | grams/1000kcal/day | |  | servings/day | |  |
|  | 33.33% | 66.67% |  | 33.33% | 66.67% |  | 33.33% | 66.67% |  | 33.33% | 66.67% |  | 33.33% | 66.67% |  | 33.33% | 66.67% |  |
| rMED | 7.66 | 10 |  | 6 | 9 |  | 5 | 7 |  | 6 | 8 |  | 7 | 9 |  | 7 | 9 |  |
| rMEDp | 7 | 10 |  | 6 | 9 |  | 5 | 7 |  | 6 | 8 |  | 7 | 9 |  | 7 | 8 |  |
| Vegetables |  |  |  |  |  |  |  |  |  |  |  |  |  |  |  |  |  |  |
| gram/day | 14.29 | 21.02 |  | 57.05 | 84.11 |  | - | - |  | - | - |  | 91.99 | 135.73 |  | - | - |  |
| serving/day | - | - |  | - | - |  | 0.36 | 0.67 |  | 0.44 | 0.71 |  | - | - |  | 1.92 | 3.12 |  |
| Legumes |  |  |  |  |  |  |  |  |  |  |  |  |  |  |  |  |  |  |
| gram/day | 4.97 | 9.01 |  | 0 | 2.5 |  | - | - |  | - | - |  | 8.99 | 17 |  | - | - |  |
| serving/day | - | - |  | - | - |  | 0 | 0.07 |  | 0 | 0.05 |  | - | - |  | 0.07 | 0.14 |  |
| Fruit |  |  |  |  |  |  |  |  |  |  |  |  |  |  |  |  |  |  |
| gram/day | 11.94 | 19.2 |  | 69.91 | 107.11 |  | - | - |  | - | - |  | 85.08 | 144.63 |  | - | - |  |
| serving/day | - | - |  | - | - |  | 0.59 | 1.08 |  | 0.86 | 1.57 |  | - | - |  | 2.57 | 3.99 |  |
| Fish |  |  |  |  |  |  |  |  |  |  |  |  |  |  |  |  |  |  |
| gram/day | 2.61 | 6.26 |  | 3.66 | 7.63 |  | - | - |  | - | - |  | 19.1 | 32.6 |  | - | - |  |
| serving/day | - | - |  | - | - |  | 0 | 0 |  | 0 | 0 |  | - | - |  | 0.21 | 0.21 |  |
| Cereals |  |  |  |  |  |  |  |  |  |  |  |  |  |  |  |  |  |  |
| gram/day | 25.14 | 37.63 |  | 83.6 | 105.41 |  | - | - |  | - | - |  | 67.17 | 88.12 |  | - | - |  |
| serving/day | - | - |  | - | - |  | 2.8 | 3.68 |  | 2.93 | 3.8 |  | - | - |  | 2.92 | 4.28 |  |
| Meat |  |  |  |  |  |  |  |  |  |  |  |  |  |  |  |  |  |  |
| gram/day | 7.77 | 11.55 |  | 31.5 | 47 |  | - | - |  | - | - |  | 54.59 | 70.7 |  | - | - |  |
| serving/day | - | - |  | - | - |  | 1.35 | 2.15 |  | 1 | 1.69 |  | - | - |  | 0.85 | 1.28 |  |
| Dairy |  |  |  |  |  |  |  |  |  |  |  |  |  |  |  |  |  |  |
| gram/day | 46.21 | 61.29 |  | 166.34 | 254.52 |  | - | - |  | - | - |  | 150.8 | 231.29 |  | - | - |  |
| serving/day | - | - |  | - | - |  | 0.66 | 1.17 |  | 0.86 | 1.27 |  | - | - |  | 2.42 | 3.72 |  |
| Olive oil |  |  |  |  |  |  |  |  |  |  |  |  |  |  |  |  |  |  |
| gram/day | 0.028 | 0.062 |  | 1.24 | 3.11 |  | - | - |  | - | - |  | 8.52 | 13.7 |  | - | - |  |
| serving/day | - | - |  | - | - |  | 0 | 0 |  | 0 | 0 |  | - | - |  | 0.07 | 0.07 |  |
| The food component “alcohol” is not presented in this table because a fixed cut-off was used. If in a cohort a food group had a very skewed distribution, the categories were differently defined, as tertiles would be impossible to calculate. In Generation R, for example, >33% of mothers reported no legumes consumption. Then the first category was zero, with >33% of the participants, the second and third categories were then defined by dividing by the median for those mothers who consumed legumes. | | | | | | | | | | | | | | | | | | |

## **Supplementary Table 3.** Epigenome-wide associations of maternal adherence to the Mediterranean diet during pregnancy (rMED) and offspring cord blood DNA methylation level for all 429,701 tested cytosine-phosphate-guanine sites, adjusted for sex + batch + cell types.

*This table is too large to include in this supplemental file, this will be made publicly available online upon acceptance of the manuscript.*

## **Supplementary Table 4.** Epigenome-wide associations of maternal adherence to the Mediterranean diet during pregnancy (rMEDp excluding the alcohol component) and offspring cord blood DNA methylation level for all 429,701 tested cytosine-phosphate-guanine sites, adjusted for sex + batch + cell types.

*This table is too large to include in this supplemental file, this will be made publicly available online upon acceptance of the manuscript.*

## **Supplementary Table 5.** Epigenome-wide associations of maternal adherence to the Mediterranean diet during pregnancy (rMED) and offspring cord blood DNA methylation level for all 429,701 tested cytosine-phosphate-guanine sites, adjusted for sex + maternal educational level + maternal age + maternal smoking + maternal BMI + maternal total energy intake + batch + cell types.

*This table is too large to include in this supplemental file, this will be made publicly available online upon acceptance of the manuscript.*

## **Supplementary Table 6.** Epigenome-wide associations of maternal adherence to the Mediterranean diet during pregnancy (rMEDp excluding the alcohol component) and offspring cord blood DNA methylation level for all 429,701 tested cytosine-phosphate-guanine sites, adjusted for sex + maternal educational level + maternal age + maternal smoking + maternal BMI + maternal total energy intake + batch + cell types.

*This table is too large to include in this supplemental file, this will be made publicly available online upon acceptance of the manuscript.*

## **Supplementary Table 7.** Cohort descriptives for the look up for association of maternal adherence to the Mediterranean diet during pregnancy with DNA methylation (cg23757341) measured in childhood.

|  | **ALSPAC** |  | **Generation R** |  | **INMA** |  | **Project Viva** |
| --- | --- | --- | --- | --- | --- | --- | --- |
| N total | 721 |  | 396 |  | 199 |  | 277 |
| rMED | 8.68 ± 2.85 |  | 8.01 ± 2.74 |  | 8.01 ± 2.65 |  | 7.91 ± 2.85 |
| rMEDp excluding alcohol | 8.54 ± 2.77 |  | 7.96 ± 2.72 |  | 7.96 ± 2.64 |  | 7.67 ± 2.82 |
|  |  |  |  |  |  |  |  |
| Age of the child at methylation measurement | 7.45 ± 0.14 |  | 6.05 ± 0.40 |  | 4.46 ± 0.22 |  | 7.77 ± 0.68 |
| Gender of the child - female | 364 (50.5%) |  | 206 (52.0%) |  | 95 (47.7%) |  | 134 (48.4%) |
| Educational level* |  |  |  |  | 49 (24.6%) |  |  |
|  | 376 (52.1%) |  | 161 (40.7%) |  | 88 (44.2%) |  | 221 (79.8%) |
|  |  |  |  |  | 62 (31.2%) |  |  |
| Maternal age at delivery (y) | 29.8 ± 4.4 |  | 32.2 ± 3.8 |  | 31.7 ± 4.2 |  | 33.9 ± 4.5 |
| Smoking* |  |  |  |  |  |  |  |
| No smoking during pregnancy |  |  | 306 (77.3%) |  | 153 (76.9%) |  |  |
| Smoked, stopped before 2^nd^ trimester | 74 (10.3%) |  | 48 (12.1%) |  | 24 (12.1%) |  | 25 (9.0%) |
| Smoked throughout pregnancy |  |  | 42 (10.6%) |  | 22 (11.1%) |  |  |
| BMI (kg/m^2^) | 22.8 ± 3.7 |  | 23.3 ± 3.8 |  | 24.4 ± 5.0 |  | 24.3 ± 4.7 |
| Total energy (kcal) | 1753 ± 453 |  | 2165 ± 506 |  | 2023 ± 487 |  | 2116 ± 604 |
| Vegetables |  |  |  |  |  |  |  |
| gram/day | 135.3 ± 68.5 |  | 162.2 ± 60.9 |  | 248.4 ± 127.5 |  | - |
| serving/day | - |  | - |  | - |  | 2.79 ± 1.47 |
| Legumes |  |  |  |  |  |  |  |
| gram/day | 53.8 ± 34.2 |  | 4.6 ± 6.6 |  | 31.6 ± 22.8 |  | - |
| serving/day | - |  | - |  | - |  | 0.16 ± 0.32 |
| Fruit and nuts |  |  |  |  |  |  |  |
| gram/day | 110.0 ± 55.4 |  | 204.9 ± 110.6 |  | 265.4 ± 172.6 |  | - |
| serving/day | - |  | - |  | - |  | 3.39 ± 1.63 |
| Fish |  |  |  |  |  |  |  |
| gram/day | 35.7 ± 28.6 |  | 13.3 ± 11.1 |  | 52.7 ± 31.3 |  | - |
| serving/day | - |  | - |  | - |  | 0.23 ± 0.19 |
| Cereals |  |  |  |  |  |  |  |
| gram/day | 236.1 ± 120.7 |  | 211.2 ± 78.0 |  | 158.5 ± 57.3 |  | - |
| serving/day | - |  | - |  | - |  | 4.15 ± 1.98 |
| Meat |  |  |  |  |  |  |  |
| gram/day | 70.2 ± 40.3 |  | 80.7 ± 37.4 |  | 129.0 ± 54.2 |  | - |
| serving/day | - |  | - |  | - |  | 1.11 ± 0.60 |
| Dairy |  |  |  |  |  |  |  |
| gram/day | 392.1 ± 157.5 |  | 477.6 ± 232.4 |  | 401.1 ± 221.6 |  | - |
| serving/day | - |  | - |  | - |  | 3.40 ± 1.54 |
| Olive oil |  |  |  |  |  |  |  |
| gram/day | 0.09 ± 0.08 |  | 5.9 ± 4.8 |  | 23.9 ± 12.4 |  | - |
| serving/day | - |  | - |  | - |  | 0.11 ± 0.30 |
| Alcohol (g/day) | 1.4 ± 3.2 |  | 0.9 ± 1.8 |  | 0.4 ± 1.5 |  | 2.29 ± 3.01 |
| Alcohol intake of 5–25 g/day | 52 (7.2%) |  | 10 (2.5%) |  | 4 (2.0%) |  | 33 (11.9%) |
| Results presented as mean ± SD or N (%) * Cohorts used their preferred categories for maternal educational level and maternal smoking during pregnancy. Please see the cohort-specific methods for these descriptions. | | | | | | | |

## **Supplementary Table 8.** Lookup of CpGs previously associated with Mediterranean diet in adults (Ma J et al. Circ Genom Precis Med 2020).

| **CpG** | **Chr** | **Gene** | **Poly-morphic** | **mQTL** | **Results from adult meta-analysis** | | | |  | **Results from our cord blood meta-analysis (rMED)** | | | |  | **Results from our cord blood meta-analysis (rMEDp)** | | | |
| --- | --- | --- | --- | --- | --- | --- | --- | --- | --- | --- | --- | --- | --- | --- | --- | --- | --- | --- |
|  |  |  |  |  | **Effect** | **SE** | **P value** | **I^2^** |  | **Effect** | **SE** | **P value** | **I^2^** |  | **Effect** | **SE** | **P value** | **I^2^** |
| cg04885881 | 1 |  | no | yes | 0.004 | 0.001 | 3.2×10^−7^ | 0.12 |  | -0.001 | 0.017 | 0.95 | 43.6 |  | -0.003 | 0.017 | 0.86 | 44.5 |
| cg19693031 | 1 | *TXNIP* | no | yes | 0.003 | 0.001 | 3.1×10^−7^ | 0.14 |  | -0.039 | 0.020 | 0.06 | 0 |  | -0.043 | 0.021 | 0.04 | 0 |
| cg01940273 | 2 |  | yes | yes | 0.005 | 0.001 | 1.6×10^−12^ | 0 |  | 0.023 | 0.017 | 0.19 | 66.3 |  | 0.023 | 0.018 | 0.19 | 66.6 |
| cg02716826 | 9 | *SUGT1P1; AQP3* | no | yes | 0.002 | 0.001 | 5.6×10^−7^ | 0 |  | 0.011 | 0.023 | 0.64 | 41.1 |  | 0.009 | 0.023 | 0.70 | 37.7 |
| cg02079413 | 11 | *SNORA54; NAP1L4* | yes | yes | −0.002 | 0.000 | 3.1×10^−7^ | 0.14 |  | -0.022 | 0.021 | 0.29 | 32.6 |  | -0.027 | 0.021 | 0.20 | 32.2 |
| cg03646329 | 13 | *LPAR6; RB1* | no | yes | 0.004 | 0.001 | 1.1×10^−6^ | 0 |  | 0.021 | 0.032 | 0.52 | 0 |  | 0.017 | 0.033 | 0.60 | 0 |
| cg16969872 | 13 | *RBM26* | yes | yes | 0.003 | 0.001 | 1.2×10^−6^ | 0.25 |  | 0.006 | 0.034 | 0.86 | 48.7 |  | 0.004 | 0.035 | 0.90 | 54 |
| cg08732950 | 16 | *CBFA2T3* | - | - | −0.003 | 0.001 | 2.8×10^−8^ | 0 |  | - | - | - | - |  | - | - | - | - |
| cg02097604 | 17 | *TOM1L2* | yes | no | 0.002 | 0.000 | 3.6×10^−8^ | 0 |  | -0.013 | 0.017 | 0.45 | 0 |  | -0.015 | 0.017 | 0.37 | 0 |
| cg18181703 | 17 | *SOCS3* | yes | yes | 0.004 | 0.001 | 3.5×10^−10^ | 0 |  | -0.006 | 0.021 | 0.76 | 26.8 |  | -0.001 | 0.021 | 0.95 | 42.1 |
| CpG: cytosine-phosphate-guanine site. Chr: chromosome. mQTL: methylation quantitative trait locus. SE: standard error. FDR: False discovery rate. | | | | | | | | | | | | | | | | | | |

## **Supplementary Table 9**. Lookup of cg23757341 in the blood autosomal cis-eQTM catalogue from the Human Early Life Exposome (HELIX) project.

| **CpG** | **Transcript Cluster** | **log2FC** | **SE** | **P value** | **sigPair** | **CpG chr** | **CpG position** | **CpG gene** | **CpG P value** | **TC gene start** | **TC gene end** | **TC gene TSS** | **TC gene** |
| --- | --- | --- | --- | --- | --- | --- | --- | --- | --- | --- | --- | --- | --- |
| cg23757341 | TC12002172.hg.1 | 0.1241 | 1.3380 | 0.35402 | FALSE | chr12 | 1725036 | *WNT5B* | 0.17503761 | 1420758 | 1422780 | 1420758 |  |
| cg23757341 | TC12001088.hg.1 | 0.1443 | 0.8328 | 0.08351 | FALSE | chr12 | 1725036 | *WNT5B* | 0.17503761 | 1489226 | 1500668 | 1500668 |  |
| cg23757341 | TC12002703.hg.1 | 0.0890 | 0.7987 | 0.26559 | FALSE | chr12 | 1725036 | *WNT5B* | 0.17503761 | 1489226 | 1500668 | 1500668 |  |
| cg23757341 | TC12002173.hg.1 | 0.0977 | 1.1450 | 0.39401 | FALSE | chr12 | 1725036 | *WNT5B* | 0.17503761 | 1526670 | 1529046 | 1526670 |  |
| cg23757341 | TC12000015.hg.1 | 0.0210 | 0.6220 | 0.73610 | FALSE | chr12 | 1725036 | *WNT5B* | 0.17503761 | 1609657 | 1613590 | 1609657 | *LINC00942* |
| cg23757341 | TC12002174.hg.1 | -0.0508 | 0.5459 | 0.35251 | FALSE | chr12 | 1725036 | *WNT5B* | 0.17503761 | 1609657 | 1616484 | 1609657 | *LINC00942* |
| cg23757341 | TC12000016.hg.1 | -0.0948 | 0.9524 | 0.31966 | FALSE | chr12 | 1725036 | *WNT5B* | 0.17503761 | 1615336 | 1616484 | 1615336 |  |
| cg23757341 | TC12001089.hg.1 | 0.0411 | 0.7483 | 0.58308 | FALSE | chr12 | 1725036 | *WNT5B* | 0.17503761 | 1609691 | 1616484 | 1616484 |  |
| cg23757341 | TC12002175.hg.1 | 0.0098 | 0.7540 | 0.89699 | FALSE | chr12 | 1725036 | *WNT5B* | 0.17503761 | 1655209 | 1655873 | 1655209 |  |
| cg23757341 | TC12000017.hg.1 | 0.0307 | 0.3828 | 0.42217 | FALSE | chr12 | 1725036 | *WNT5B* | 0.17503761 | 1683823 | 1756409 | 1683823 | *WNT5B* |
| cg23757341 | TC12001090.hg.1 | -0.1387 | 0.9990 | 0.16533 | FALSE | chr12 | 1725036 | *WNT5B* | 0.17503761 | 1675159 | 1703331 | 1703331 | *FBXL14* |
| cg23757341 | TC12002704.hg.1 | 0.0505 | 0.6626 | 0.44579 | FALSE | chr12 | 1725036 | *WNT5B* | 0.17503761 | 1762424 | 1778165 | 1778165 |  |
| cg23757341 | TC12000018.hg.1 | -0.2216 | 0.9014 | 0.01418 | FALSE | chr12 | 1725036 | *WNT5B* | 0.17503761 | 1800193 | 1897845 | 1800193 | *ADIPOR2* |
| cg23757341 | TC12000019.hg.1 | -0.0762 | 0.3688 | 0.03921 | FALSE | chr12 | 1725036 | *WNT5B* | 0.17503761 | 1929433 | 1945918 | 1929433 | *LRTM2* |
| cg23757341 | TC12002176.hg.1 | 0.0632 | 1.0894 | 0.56232 | FALSE | chr12 | 1725036 | *WNT5B* | 0.17503761 | 2027014 | 2029505 | 2027014 | *CACNA2D4* |
| cg23757341 | TC12000020.hg.1 | 0.0215 | 0.8909 | 0.80975 | FALSE | chr12 | 1725036 | *WNT5B* | 0.17503761 | 2027117 | 2032033 | 2027117 |  |
| cg23757341 | TC12001092.hg.1 | -0.0112 | 0.2642 | 0.67119 | FALSE | chr12 | 1725036 | *WNT5B* | 0.17503761 | 1901123 | 2028002 | 2028002 | *CACNA2D4* |
| cg23757341 | TC12002705.hg.1 | -0.0950 | 0.5092 | 0.06256 | FALSE | chr12 | 1725036 | *WNT5B* | 0.17503761 | 2038368 | 2045740 | 2045740 | *LINC00940* |
| cg23757341 | TC12001093.hg.1 | -0.0816 | 0.8694 | 0.34821 | FALSE | chr12 | 1725036 | *WNT5B* | 0.17503761 | 2038368 | 2045742 | 2045742 | *LINC00940* |
| cg23757341 | TC12000021.hg.1 | -0.0629 | 0.9341 | 0.50070 | FALSE | chr12 | 1725036 | *WNT5B* | 0.17503761 | 2080229 | 2080366 | 2080229 | *CACNA1C* |
| cg23757341 | TC12001094.hg.1 | 0.0324 | 0.6587 | 0.62245 | FALSE | chr12 | 1725036 | *WNT5B* | 0.17503761 | 2050757 | 2113701 | 2113701 | *DCP1B* |
| cg23757341 | TC12000022.hg.1 | 0.0209 | 0.7197 | 0.77194 | FALSE | chr12 | 1725036 | *WNT5B* | 0.17503761 | 2113832 | 2120558 | 2113832 |  |
| cg23757341 | TC12000024.hg.1 | -0.0855 | 1.0511 | 0.41603 | FALSE | chr12 | 1725036 | *WNT5B* | 0.17503761 | 2157518 | 2158629 | 2157518 | *CACNA1C-IT2* |
| cg23757341 | TC12000025.hg.1 | 0.0121 | 0.1621 | 0.45413 | FALSE | chr12 | 1725036 | *WNT5B* | 0.17503761 | 2162416 | 2807115 | 2162416 | *CACNA1C* |
| CpG: cytosine-phosphate-guanine site. log2FC = change in expression as log2 fold change per 10% DNA methylation. P value = P value of the CpG-TC association. sigPair = is the pair significant after multiple testing correction (also for the number of TCs each CpG is paired with)? TRUE/FALSE. CpG chr = Chromosome  CpG P value = P value that the CpG is an eQTM. TC gene start = start position of the Transcript Cluster (gene). TC gene end = end position of the Transcript Cluster (gene). TC gene TSS = position used as Transcription Start Site for the Transcript Cluster (gene). TC gene = genes annotated to Transcript Cluster (gene) according to Affymetrix na36 annotation. | | | | | | | | | | | | | |

## **Supplementary Table 10.** Gene ontology (GO) results (P<0.01), based on n=81 CpGs with P<0.0001.

| **ONTOLOGY** | **TERM** | **N** | **DE** | **P value** | **FDR** |
| --- | --- | --- | --- | --- | --- |
| GO BP | UTP metabolic process | 11 | 2 | 3.40E-04 | 1 |
| GO BP | pyrimidine ribonucleoside triphosphate metabolic process | 15 | 2 | 7.60E-04 | 1 |
| GO BP | pyrimidine nucleoside triphosphate metabolic process | 21 | 2 | 1.64E-03 | 1 |
| GO BP | hyaluronan biosynthetic process | 14 | 2 | 1.78E-03 | 1 |
| GO BP | pyrimidine ribonucleotide metabolic process | 22 | 2 | 2.07E-03 | 1 |
| GO BP | GTP metabolic process | 24 | 2 | 2.22E-03 | 1 |
| GO BP | N-terminal peptidyl-L-cysteine N-palmitoylation | 1 | 1 | 2.31E-03 | 1 |
| GO BP | phosphatidylethanolamine catabolic process | 2 | 1 | 4.72E-03 | 1 |
| GO BP | protein palmitoylation | 31 | 2 | 4.92E-03 | 1 |
| GO BP | prostanoid metabolic process | 41 | 2 | 5.13E-03 | 1 |
| GO BP | prostaglandin metabolic process | 41 | 2 | 5.13E-03 | 1 |
| GO BP | N-terminal protein palmitoylation | 2 | 1 | 5.18E-03 | 1 |
| GO BP | ITP metabolic process | 2 | 1 | 5.35E-03 | 1 |
| GO BP | glucose import in response to insulin stimulus | 2 | 1 | 5.60E-03 | 1 |
| GO BP | metanephric glomerular mesangial cell development | 1 | 1 | 6.48E-03 | 1 |
| GO BP | guanosine-containing compound metabolic process | 36 | 2 | 6.54E-03 | 1 |
| GO BP | glycosyl compound metabolic process | 120 | 3 | 6.90E-03 | 1 |
| GO BP | cAMP metabolic process | 22 | 2 | 7.19E-03 | 1 |
| GO BP | positive regulation of vascular smooth muscle cell dedifferentiation | 2 | 1 | 7.58E-03 | 1 |
| GO BP | regulation of DNA strand resection involved in replication fork processing | 1 | 1 | 7.61E-03 | 1 |
| GO BP | purine ribonucleoside monophosphate metabolic process | 33 | 2 | 8.02E-03 | 1 |
| GO BP | organophosphate metabolic process | 973 | 9 | 8.26E-03 | 1 |
| GO BP | purine nucleoside monophosphate metabolic process | 34 | 2 | 8.36E-03 | 1 |
| GO BP | response to jasmonic acid | 4 | 1 | 8.70E-03 | 1 |
| GO BP | cellular response to jasmonic acid stimulus | 4 | 1 | 8.70E-03 | 1 |
| GO BP | regulation of complement activation | 50 | 2 | 8.96E-03 | 1 |
| GO BP | regulation of 1-phosphatidylinositol 4-kinase activity | 1 | 1 | 9.04E-03 | 1 |
| GO BP | positive regulation of 1-phosphatidylinositol 4-kinase activity | 1 | 1 | 9.04E-03 | 1 |
| GO BP | positive regulation of adaptive immune memory response | 1 | 1 | 9.04E-03 | 1 |
| GO BP | metanephric mesangial cell differentiation | 2 | 1 | 9.18E-03 | 1 |
| GO BP | metanephric glomerular mesangial cell differentiation | 2 | 1 | 9.18E-03 | 1 |
| GO BP | hyaluronan metabolic process | 36 | 2 | 9.32E-03 | 1 |
| GO BP | response to mycophenolic acid | 2 | 1 | 9.33E-03 | 1 |
| GO BP | cellular response to mycophenolic acid | 2 | 1 | 9.33E-03 | 1 |
| GO BP | nucleoside phosphate metabolic process | 511 | 6 | 9.36E-03 | 1 |
| GO CC | meprin A complex | 2 | 1 | 4.08E-03 | 1 |
| GO CC | immunological synapse | 36 | 2 | 9.12E-03 | 1 |
| GO MF | chlordecone reductase activity | 1 | 1 | 2.39E-03 | 1 |
| GO MF | calcium- and calmodulin-responsive adenylate cyclase activity | 2 | 1 | 7.17E-03 | 1 |
| GO MF | 5'-3' exoribonuclease activity | 3 | 1 | 7.24E-03 | 1 |
| GO MF | thyroxine 5'-deiodinase activity | 3 | 1 | 7.90E-03 | 1 |
| GO MF | nucleoside triphosphate adenylate kinase activity | 2 | 1 | 8.47E-03 | 1 |
| GO MF | nucleobase-containing compound kinase activity | 41 | 2 | 9.25E-03 | 1 |
| GO MF | catalytic activity | 5255 | 28 | 9.30E-03 | 1 |
| GO MF | FFAT motif binding | 3 | 1 | 9.82E-03 | 1 |

# Supplementary cohort-specific methods

## ***The Avon Longitudinal Study of Parents and Children (ALSPAC)***

**Description of cohort**:

Pregnant women resident in Avon, UK with expected dates of delivery 1st April 1991 to 31st December 1992 were invited to take part in the study (1,2). The initial number of pregnancies enrolled is 14,541 (for these at least one questionnaire has been returned or a “C_h_i_l_d_r_e_n_ _i_n_ _F_o_c_u_s_” _c_l_i_n_i_c_ _had been attended by 19/07/99). Of these initial pregnancies, there was a total of 14,676 foetuses, resulting in 14,062 live births and 13,988 children who were alive at 1 year of age.

When the oldest children were approximately 7 years of age, an attempt was made to bolster the initial sample with eligible cases who had failed to join the study originally. As a result, when considering variables collected from the age of seven onwards (and potentially abstracted from obstetric notes) there are data available for more than the 14,541 pregnancies mentioned above. The number of new pregnancies not in the initial sample (known as Phase I enrolment) that are currently represented on the built files and reflecting enrolment status at the age of 24 is 913 (456, 262 and 195 recruited during Phases II, III and IV respectively), resulting in an additional 913 children being enrolled. The phases of enrolment are described in more detail in the cohort profile paper and its update. The total sample size for analyses using any data collected after the age of seven is therefore 15,454 pregnancies, resulting in 15,589 foetuses. Of these 14,901 were alive at 1 year of age.

A 10% sample of the ALSPAC cohort, known as the Children in Focus (CiF) group, attended clinics at the University of Bristol at various time intervals between 4 to 61 months of age. The CiF group were chosen at random from the last 6 months of ALSPAC births (1432 families attended at least one clinic). Excluded were those mothers who had moved out of the area or were lost to follow-up, and those partaking in another study of infant development in Avon. As part of the Accessible Resources for Integrated Epigenomic Studies (ARIES (3)) project, DNA methylation was generated for 1018 mother-offspring pairs from the ALSPAC cohort, using the Infinium HumanMethylation450 BeadChip array (Illumina Inc., San Diego, United States). ARIES participants were selected based on availability of DNA samples at two time points for the mother (antenatal and at follow-up when the offspring were adolescents) and at three time points for the offspring (neonatal, childhood (age 7), and adolescence (age 15/17)).

**Ethical approval:**

Ethical approval for the study was obtained from the ALSPAC Ethics and Law Committee and the Local Research Ethics Committees. Initial approval references are: [1] Bristol and Weston Health Authority: E1808 Children of the Nineties: Avon Longitudinal Study of Pregnancy and Childhood (ALSPAC). (28th November 1989); [2] Southmead Health Authority: 49/89 Children of the Nineties -"ALSPAC". (5th April 1990); [3] Frenchay Health Authority: 90/8 Children of the Nineties. (28th June 1990). Consent for biological samples has been collected in accordance with the Human Tissue Act (2004). Informed consent for the use of data collected via questionnaires and clinics was obtained from the participants following the recommendations of the ALSPAC Ethics and Law Committee at the time.

**EWAS analyst:**

Aayah Nounu

**Description of detail in FFQ:**

We used a 47-item (of which 42 items were used for this study) food frequency questionnaire (FFQ) with questions worded as “How many times nowadays do you eat [*food item*]?” (4). Maternal nutritional intake was registered around 32 weeks of gestation with the reference period being “nowadays”. The FFQ was validated for measuring oily fish intake (5). **Vegetables** included leafy green, carrots, salad, other green and other root vegetables. **Legumes** included baked beans, legumes and pulses. **Fruit/Nuts/Seeds** included fresh fruit, nuts and tahini. **Fish** included white fish, oily fish and shellfish. **Cereals** included bread (white, brown, wholemeal or chapati), rice, pasta, pizza, oat cereals, bran cereal, crispbread and “others, if consumed cereal type was not listed. **Meat** included sausages, burgers, red meat, poultry, offal, pies and pasties. **Dairy** included milk (in tea, coffee, with cereal, pudding and on its own), milky drink and cheese. **Olive oil** included oil for frying. **Alcohol** was based on total alcohol intake.

**Methylation normalization and QC steps taken in your cohort:**

*Cord blood samples:*

Methods for methylation measurements in ALSPAC have been described previously (3). Briefly, cord blood was collected according to standard procedures. DNA methylation assays and data pre-processing were performed at the University of Bristol as part of the ARIES project. DNA was extracted using standard protocol and was bisulfite-converted using the Zymo EZ DNA MethylationTM kit (Zymo, Irvine, CA). DNA methylation was then measured using the Infinium HM450 BeadChip assay (Illumina Inc, San Diego, CA), according to the standard protocol. Arrays were scanned using an Illumina iScan. An initial review of data quality was assessed using GenomeStudio (version 2011.1). A semi-random approach (sampling criteria were in place to ensure that all time points were represented on each array) was used to distribute ARIES samples across slides to minimize the possibility of potential confounding by batch. Data were normalised using the meffil R package (6) using the functional normalisation approach.

In this study, methylation outliers were removed using the IQR*3 (Tukey) method and probes with high detection p-values were removed.

*Childhood blood samples:*

For the look-up in childhood, we used DNA extracted from blood samples taken at the 7-year follow-up visit. The procedure for measuring and normalization of methylation data, and QC steps were similar to that for the cord blood samples.

**Covariates:**

**Maternal education** was defined based on the UK highest qualification achieved by the mothers. The covariate is binary and CSE/none, Vocational and O-level qualifications were grouped to describe lower educational level and A-level and university degree to describe higher educational level. **Maternal smoking** during pregnancy was determined by questionnaire at the time of recruitment and defined here as “no smoker” if mother was a never smoker or quit before second trimester, or as a “smoker” if mother smoked and did not quit before second trimester. **Newborn sex** was obtained from obstetric records. **Maternal age** was derived from the mother’s date of birth at the time of delivery. **Maternal BMI** was calculated from self-reported height and pre-pregnancy weight, which were collected by questionnaire during the first trimester of pregnancy. **Child ethnic group** was derived from mother reports of her own and partners ethnic group during pregnancy. Participants with non-white European ancestry were excluded from all analyses. **Cord blood** **cell type composition** was estimated using the “Salas” reference set (7) in the ‘’FlowSorted.CordBlood.Combined.450K’’ Bioconductor package for cell type correction and normalised using meffil R package (6). We attempted to control for technical **batch effects** by generating 20 surrogate variables using the SVA R package (8) and including these directly in the EWAS models.

For the **look-up in childhood** (mean age 7.4y), we additionally adjusted for **childhood age** at DNA methylation measurement. Further, for this look-up in childhood, **cell type correction** was applied using estimates calculated using the Houseman method (9) .

**Funding:**

GM and GCS are members of the MRC Integrative Epidemiology Unit, which receives funds from the University of Bristol and United Kingdom Medical Research Council [MC_UU_00011/1, MC_UU_00011/5 and MC_UU_00011/6]. GCS’s contribution to this work is supported by the Medical Research Council [New Investigator Research Grant, MR/S009310/1]. GM, LJ and GCS’s contributions are supported by the European Joint Programming Initiative “A Healthy Diet for a Healthy Life” (JPI HDHL, NutriPROGRAM project, UK MRC MR/S036520/1]. AN’s contribution to this work is supported by Cancer Research UK (C18281/A19169) The UK Medical Research Council and Wellcome (Grant ref: 217065/Z/19/Z) and the University of Bristol provide core support for ALSPAC. A comprehensive list of grants funding is available on the ALSPAC website (http://www.bristol.ac.uk/alspac/external/documents/grant-acknowledgements.pdf). The Accessible Resource for Integrated Epigenomics Studies (ARIES) which generated large scale methylation data was funded by the UK Biotechnology and Biological Sciences Research Council (BB/I025751/1 and BB/I025263/1). Additional epigenetic profiling on the ALSPAC cohort was supported by the UK Medical Research Council Integrative Epidemiology Unit and the University of Bristol (MC_UU_12013_1, MC_UU_12013_2, MC_UU_12013_5 and MC_UU_12013_8), the United States National Institute of Health (5RO1AI121226-02) and National Institute of Child and Human Development grant (R01HD068437). The funders had no role in study design, data collection and analysis, decision to publish, or preparation of the manuscript. This publication is the work of the authors and Gemma Sharp will serve as guarantors for the contents of this paper. The views expressed in this paper are those of the authors and not necessarily any funders. The funders had no influence on the content of the paper.

**Acknowledgements:**

We are extremely grateful to all the families who took part in this study, the midwives for their help in recruiting them, and the whole SWS and ALSPAC teams, which includes interviewers, computer and laboratory technicians, clerical workers, research scientists, volunteers, managers, receptionists, and nurses. Please note that the ALSPAC study website (http://www.bristol.ac.uk/alspac/researchers/our-data/) contains details of all the data that is available through a fully searchable data dictionary and variable search tool.

## ***The Generation R Study***

**Description of cohort**:

The Generation R Study is a prospective population-based cohort in Rotterdam, the Netherlands (10,11). All pregnant women residing in Rotterdam with a delivery date between April 2002 and January 2006 were invited to participate. The Medical Ethical Committee of Erasmus MC, University Medical Center Rotterdam, approved the study and an informed consent was obtained for all participating children. In total, 9,778 mothers were enrolled in the Generation R study. The present analyses were limited to mothers of Dutch national origin, since nutrition generally differs between ethnic groups and the FFQ was validated for the assessment of dietary intake in a Dutch population.

**Ethical approval:**

The study was conducted in accordance with the guidelines proposed in the World Medical Association Declaration of Helsinki and was approved (MEC-198.782.2001.31) by the Medical Ethics Committee at Erasmus Medical Center, University Medical Center Rotterdam, The Netherlands. Written consent was obtained from all participants.

**EWAS analyst:**

Leanne Küpers

**Description of detail in FFQ:**

We used a modified version of a validated semi-quantitative 293-item food frequency questionnaire (FFQ) (12). Maternal nutritional intake was registered around median 13.5 weeks of gestation, 95% range 10.1-21.8 weeks, with the past three months as the reference period. **Vegetables** included leafy vegetables, fruit vegetables, root vegetables, cabbages, mushrooms, onions and garlic, stem vegetables, sprout vegetables, mixed salads/vegetables. **Legumes** included one question on legumes. We observed a skewed distribution for legumes consumption (with a large portion of the cohort never consuming them), thus we set the first category to zero, where this first category includes >33% of the participants. The second and third categories are then defined by dividing by the median for those mothers who consumed >0 g/day. This is done according to the R script provided in the analysis plan. **Fruit/Nuts/Seeds** included fruits, nuts, seeds, nut spread, mixed fruits/nuts. **Fish** included lean fish, fatty fish, fish products, fish sticks. **Cereals** included flour, flakes, binders, pasta, rice, other grains, bread, knackebrot, rusk, breakfast cereals, pretzels, toast. **Meat** included beef, veal, pork, mutton/lamb, horse, poultry, meat products, organ meat. **Dairy** included milk, milk drinks, yogurt, quark, fresh cheese, cheese, puddings with a base of milk/mousse/cream, cream, cream substitutes, coffee creamer, creamers. **Olive oil** included one question on olive oil. **Alcohol** was based on total alcohol intake.

**Methylation normalization and QC steps taken in your cohort:**

*Cord blood samples:*

DNA extracted (using the salting-out method) from blood samples taken at birth (cord blood) was used for this analysis. 500 ng DNA per sample underwent bisulfite conversion using the EZ-96 DNA Methylation kit (Shallow) (Zymo Research Corporation, Irvine, USA). Samples were plated onto 96-well plates in no specific order. Samples were processed with the Illumina Infinium HumanMethylation450 BeadChip (Illumina Inc., San Diego, USA). Preparation and normalization of the HumanMethylation450 BeadChip array data was performed according to the CPACOR workflow (13) using the software package R (14). In detail, the idat files were read using the minfi package. Probes that had a detection p-value above background (based on sum of methylated and unmethylated intensity values) ≥ 1E-16 were set to missing per array. Next, the intensity values were stratified by autosomal and non-autosomal probes and quantile normalized for each of the six probe type categories separately: type II red/green, type I methylated red/green and type I unmethylated red/green. Beta values were calculated as proportion of methylated intensity value on the sum of methylated+unmethylated+100 intensities. Arrays with observed technical problems such as failed bisulfite conversion, hybridization or extension, as well as arrays with a mismatch between sex of the proband and sex determined by the chr X and Y probe intensities were removed from subsequent analyses. Additionally, only arrays with a call rate > 95% per sample were processed further. Probes on the X and Y chromosomes were excluded from the dataset. The final dataset contained information on 458,563 CpGs.

*Childhood blood samples:*

For the look-up in childhood, we used DNA extracted (using the salting-out method) from blood samples taken at the 5-year follow-up visit. The procedure for processing with the Illumina Infinium HumanMethylation450 BeadChip (Illumina Inc., San Diego, USA) and for the normalization and QC steps was similar to that for the cord blood samples.

**Covariates:**

**Maternal education** was self-reported and defined based on the highest level of education finished by the mothers. The covariate is binary with higher education describing the higher educational level. **Maternal smoking** during pregnancy was self-reported as “no smoking during pregnancy”, “smoked but quit before second trimester”, or “smoked throughout pregnancy”. **Newborn sex** was obtained from midwife and hospital registries. **Maternal age** was reported by the mother in the questionnaire. **Maternal BMI** was calculated based on self-reported height and weight. If available, pre-pregnancy BMI was used, otherwise BMI in early pregnancy was used. **Maternal total energy intake** was calculated in kcal/day by means of the self-reported FFQ and the Dutch food composition table. **Cell type composition** was estimated using the reference-based Houseman method (9) in the minfi Package (15) in R (14). This method estimates the relative proportions of white blood cell subtypes CD8T, CD4T, NK, Bcells, Monocytes, Granulocytes, nucleated red blood cells in cord blood (7). Plate number was included as **batch variable**.

For the **look-up in childhood** (mean age 6y), we additionally adjusted for **childhood age** at DNA methylation measurement. Further, for this look-up in childhood, **cell type correction** was applied using the reference-based Houseman method (9) in the minfi package (15) in R (14), using the adult Reinius reference panel (16). This method estimates the relative proportions of six white blood cell subtypes (CD4+ T-lymphocytes, CD8+ T-lymphocytes, NK (natural killer) cells, B-lymphocytes, monocytes and granulocytes), based on a standard reference population (as published by Reinius *et al*.) (16).

**Funding:**

The general design of the Generation R Study is made possible by financial support from the Erasmus Medical Center, Rotterdam, the Erasmus University Rotterdam, the Netherlands Organization for Health Research and Development and the Ministry of Health, Welfare and Sport. The EWAS data was funded by a grant to VWJ from the Netherlands Genomics Initiative (NGI)/Netherlands Organisation for Scientific Research (NWO) Netherlands Consortium for Healthy Aging (NCHA; project nr. 050-060-810), by funds from the Genetic Laboratory of the Department of Internal Medicine, Erasmus MC, and by a grant from the National Institute of Child and Human Development (R01HD068437). VWJ received an additional grant from the Netherlands Organization for Health Research and Development (VIDI 016.136.361) and a Consolidator Grant from the European Research Council (ERC-2014-CoG-648916). This project received funding from the European Union’s Horizon 2020 research and innovation programme (733206, LifeCycle; 874739, LongITools; 824989, EUCAN-Connect) and from the European Joint Programming Initiative “A Healthy Diet for a Healthy Life” (JPI HDHL, NutriPROGRAM project, ZonMw the Netherlands no.529051022 and JPI HDHL, PREcisE project, ZonMw, the Netherlands no.529051023).

**Acknowledgements:**

The Generation R Study is conducted by the Erasmus Medical Center in close collaboration with the School of Law and Faculty of Social Sciences of the Erasmus University Rotterdam, the Municipal Health Service Rotterdam area, Rotterdam, the Rotterdam Homecare Foundation, Rotterdam and the Stichting Trombosedienst & Artsenlaboratorium Rijnmond (STAR-MDC), Rotterdam. We gratefully acknowledge the contribution of children and parents, general practitioners, hospitals, midwives and pharmacies in Rotterdam. The study protocol was approved by the Medical Ethical Committee of the Erasmus Medical Centre, Rotterdam. Written informed consent was obtained for all participants. The generation and management of the Illumina 450K methylation array data (EWAS data) for the Generation R Study was executed by the Human Genotyping Facility of the Genetic Laboratory of the Department of Internal Medicine, Erasmus MC, the Netherlands. We thank Mr. Michael Verbiest, Ms. Mila Jhamai, Ms. Sarah Higgins, Mr. Marijn Verkerk and Dr. Lisette Stolk for their help in creating the EWAS database. We thank Dr. A.Teumer for his work on the quality control and normalization scripts.

## ***Healthy Start***

**Description of cohort**:

Healthy Start is an ongoing, longitudinal pre-birth cohort study that enrolled 1,410 pregnant women in Colorado, USA between 2009-2014 (17,18). Eligible women were at least 16 years old, had singleton pregnancies at <24 weeks of gestation at the time of enrollment, and no serious chronic medical conditions or history of stillbirth or extremely preterm birth. Cord blood was collected at delivery and DNA was extracted for methylation analysis.

**Ethical approval:**

The Healthy Start study protocol was approved by the Colorado Multiple Institutional Review Board, protocol #09-0563, and all women provided written informed consent before the first study visit.

**EWAS analyst:**

Anne Starling

**Description of detail in FFQ:**

We used the validated Automated Self-Administered 24-hour dietary Assessment Tool (ASA24) (19). Participants completed a mean of 2.8 recalls (SD 1.6), and a median of 2.0 recalls (range 1-8). Maternal nutritional intake was registered around median 25 weeks of gestation, range 9.7-39.9 weeks. **Vegetables** included dark-green vegetables, orange vegetables, other starchy vegetables, tomatoes, other vegetables, measured in cup equivalents. **Legumes** included cooked dry beans and peas, measured in cup equivalents. **Fruit/Nuts/Seeds** included citrus fruits, melons, and berries, other fruits, nuts and seeds, measured in cup equivalents. **Fish** included fish and shellfish high in n-3 fatty acids, fish and shellfish low in n-3 fatty acids, measured in ounce equivalents. **Cereals** included whole and non-whole/refined grains. **Meat** included meat (beef, pork, veal, lamb, game), organ meats (meat, poultry), frankfurters, sausage, luncheon meats (made from meat or poultry), poultry (chicken, turkey, other), measured in ounce equivalents. **Dairy** included Milk, yogurt, cheese, measured in cup equivalents. **Olive oil** was based on total olive oil consumption, measured in grams. **Alcohol** was based on total alcohol intake, measured in grams.

**Additional information regarding the construction of the Mediterranean diet scores:**

In Healthy Start cup/oz equivalents were used instead of grams. 24-hour recalls were used and averages of consumptions were taken across available intakes. When creating the derived food components, we sometimes had multiple sub-components. For example, for the vegetable food component, we summed across dark green vegetables, orange vegetables, starchy vegetables (other than white potatoes), tomatoes, and other vegetables. We first averaged across all intake days to get an average intake for the sub-component. We then summed the averages to get the vegetable food component. **Fruit/nuts/seeds**- Combined the total fruit (cup equivalents) with the nuts and seeds (oz. equivalents of lean meat) variables. Note that by combining these two variables, the units now have no meaning. This will occur consistently across all participants, and we are combining them into cohort based tertiles, therefore this should not cause problems. **Vegetables**- There were three participants where vegetable consumption was blank at the intake level, these values have been set to 0, and then averages were taken. **Legumes, fish, and olive oil** all had skewed distributions (with a large portion of the cohort never consuming them), thus we set the first category to zero, where this first category includes >33% of the participants. The second and third categories are then defined by dividing by the median for those mothers who consumed >0 g/day. This is done according to the R script provided in the analysis plan. There are participants with values between 0.00 and 0.01 for the proportion of the calories consumed. Note: R code altered to include any non-zero value in the calculation of the median.

**Methylation normalization and QC steps taken in your cohort:**

DNA extracted from cord blood was used for this analysis. We removed 587 probes with high detection P value (P>0.05). We removed 664 probes with a beadcount < 3 in at least 5% of samples. We compared the predicted sex to the reported sex. Any samples with inconsistent sex were removed (n=5). preprocessQuantile function in Minfi (15) was used to normalize. ComBat was used for batch correction, followed by adjustment for Sample_Plate within models. Methylation outliers were removed using the 3*IQR method. Eligibility criteria: Healthy Start enrolled only singleton births (no multiples). Of 1410 enrolled participants, 600 had DNA methylation in cord blood assessed via the Illumina 450K array. Of these, 8 were excluded for missing dietary intake data in pregnancy and 77 were excluded for missing smoking data. Siblings were excluded at random (n=7). Participants for whom reported and predicted sex did not match were excluded (n=5). Major racial/ethnic groups were analyzed separately, and only the Hispanic and non-Hispanic white participants had sufficient sample size to run the EWAS. The sample sizes were 131 for Hispanic and 257 for non-Hispanic white.

**Covariates:**

**Maternal education** was self-reported and defined based on the highest level of education finished by the mothers. The covariate is binary with high describing more than high school and low describing high school or less. **Maternal smoking** during pregnancy was self-reported at three time points during pregnancy and subsequently classified as “no smoking during pregnancy or stopped early in pregnancy”, or “sustained smoking during pregnancy”. **Newborn sex** was obtained from medical records. **Maternal age** was self-reported at the time of study enrollment. **Maternal BMI** was calculated based height and weight. Height measured at first study visit and weight obtained from medical record or self-report at study enrollment if unavailable in the medical record. **Maternal total energy intake** was calculated in kcal/day by means of the ASA24. **Cell type composition** in umbilical cord blood (relative proportions of B cells, CD4 T cells, CD8 T cells, granulocytes, monocytes, NK cells, and nucleated red blood cells) was estimated using estimateCellCounts2 function in the R package FlowSorted (R version 3.6.2, R Foundation for Statistical Computing). CordBloodCombined.450k, using a combined cord blood reference data set (7). ComBat was used for batch correction (8), followed by adjustment for Sample_Plate within models as **batch variable**.

**Funding:**

Anne P Starling and Chloe Friedman were funded by a grant from the National Institute of Environmental Health Sciences (R00ES025817). Dana Dabelea was funded by National Institute of Diabetes and Digestive and Kidney Diseases (R01DK076648), and the National Institutes of Health Office of the Director (UH3OD023248).

**Acknowledgements:**

None.

## ***The INMA—INfancia y Medio Ambiente—(Environment and Childhood) Project***

**Description of cohort**:

The present study used data from participants recruited between 2003 and 2008 in the de novo cohort sited in Sabadell of the INfancia y Medio Ambiente (INMA) Project, a population-based mother–child cohort study in Spain (20). Current project uses data from European ancestry children from the Sabadell subcohort. Study website: http://www.proyectoinma.org/

**Ethical approval:**

INMA Sabadell was approved by the Ethics Committee ”Comité Ético de Investigación Clínica del Instituto Municipal de Asistencia Sanitaria” under project number 2005/2106/I, project name “INMA-infancia y Medio Ambiente- Barcelona-IMIM”. All participants gave their written informed consent.

**EWAS analyst:**

Sílvia Fernández-Barrés

**Description of detail in FFQ:**

We used a biomarker-validated 101-item FFQ and standard units and serving sizes were specified for each food item (21). Maternal nutritional intake was registered in the first trimester around 12 weeks of gestation, with the first trimester as the reference period. **Vegetables** included vegetables and tomato sauce. **Legumes** included all legumes. **Fruit/Nuts/Seeds** included oranges, bananas, apples, peach, water melon, grapes, dry fruits, kiwi, fresh orange juice, nuts. **Fish** included fried fish, lean fish, fatty fish (small and big), smoked fish, canned fish, seafood (shellfish, squid). **Cereals** included cereals, rice, pasta, corn. **Meat** included chicken, veil, pork, rabbit, liver, entrails, cold meats, sausages, pates, burgers, bacon. **Dairy** included milk (all types), cream, yogurt, cheese (all types), pudding/custard, ice creams. **Olive oil** included one question on olive oil. **Alcohol** was based on total alcohol intake in grams.

**Methylation normalization and QC steps taken in your cohort:**

*Cord blood and childhood blood samples:*

DNA from cord blood or child blood (collected at the age of 4y) was extracted using the Chemagen kit (Perkin Elmer). DNA concentration was determined by NanoDrop spectrophotometer (Thermo Scientific) and with the Quant-iT PicoGreen dsDNA Assay Kit (Life Technologies). Methylation data was produced in two different laboratories as part of two different projects: in the Genome Analysis Facility of the University Medical Center Groningen (UMCG) in Holland (cord blood and child blood), and in the Bellvitge Biomedical Research Institute (IDIBELL, Barcelona) (additional cord blood samples). Both laboratories used the recommended Illumina protocol for the Infinium HumanMethylation450 beadchip. Briefly, 500 ng of DNA was bisulfite-converted using the EZ 96-DNA methylation kit following the manufacturer’s standard protocol, and DNA methylation measured using the Illumina Infinium HumanMethylation450 beadchip. DNA methylation data were quality controlled and preprocessed using the minfi package (15). A series of steps were completed for quality control and data analysis. The first step was low quality sample removal. First, 2 samples with bad overall quality or with low detection p-value according to the output of the MethylAid package (22) were removed. Then, we removed 3 samples whose sex was wrongly predicted using shinyMethyl were eliminated (23). Following guidelines of Lehne work (13), we increased the stringency of the detection p-value threshold to 10E-16 and we filtered 18 samples with a call rate lower than 98%. Data was normalized with the functional normalization method. Correlation between SNPs in replicates replicated samples was checked and probes not measuring SNPs were discarded. 7,136 probes with a call rate lower than 95% were also removed. Probes in sexual chromosomes, cross-hybridizing or containing SNPs were flagged but not removed at this point. ComBat was applied to remove laboratory batch effect (24). Finally, duplicated samples were removed, prioritizing MeDALL samples over BREATHE samples. The final dataset consisted of 476,946 probes and 380 (European ancestry and with exposure data) at age 0y and; and 199 (European ancestry and with exposure data) at age 4y.

**Covariates**

**Maternal education** was assessed in week 12 of gestation and coded as an ordinal variable: 1 = primary or without education, 2= secondary, and 3 = University. **Maternal smoking** was assessed as an ordinal variable representing 1 = No smoking during pregnancy, 2 = Smoked, stopped before 2nd trimester, 3 = Smoked throughout pregnancy. **Newborn sex** was taken from obstetric records. **Maternal age** was a continuous numeric variable in years assessed at enrolment. **Maternal pre-pregnancy BMI** was calculated from measured height and self-reported pre-pregnancy weight collected using a questionnaire at enrolment (week 12 of pregnancy). Reported pre-pregnancy weight was highly correlated with measured weight at 12 weeks of pregnancy in INMA (r= 0.96; P < 0.0001). **Maternal total energy intake** was assessed in the first trimester of pregnancy using a 101-item FFQ. Total energy intake was obtained from the US Department of Agriculture food composition tables and other published sources (25,26). **Cord blood cell proportions** were estimated using the Gervin and Salas reference panel (7), the IDOL algorithm for selection of 517 CpGs (for 450K and EPIC arrays) (27), and the constrained projection-quadratic programming algorithm by Houseman (9) for deconvolution of 7 main blood cell types. Combat was used as laboratory **batch variable**.

For the **look-up in childhood** (mean age 4.46y), we additionally adjusted for **childhood age** at DNA methylation measurement. Further, for this look-up in childhood, **cell type correction** was applied using the Reinius reference panel (16) with the pickCompProbes method (minfi (15)) for CpG selection, and the Houseman algorithm (9) for deconvolution of 6 main blood cell types.

**Funding:**

This study was funded by grants from Instituto de Salud Carlos III (Red INMA G03/176; CB06/02/0041; PI041436; PI081151 incl. FEDER funds), Generalitat de Catalunya-CIRIT 1999SGR 00241, Fundació La marató de TV3 (090430), EU Commission (261357-MeDALL: Mechanisms of the Development of ALLergy), and European Research Council (268479-BREATHE: BRain dEvelopment and Air polluTion ultrafine particles in scHool childrEn). We acknowledge support from the Spanish Ministry of Science and Innovation and the State Research Agency through the “Centro de Excelencia Severo Ochoa 2019-2023” Program (CEX2018-000806-S), and support from the Generalitat de Catalunya through the CERCA Program.

**Acknowledgements:**

We particularly thank all the cohort participants for their generous collaboration.

## ***Project Viva***

**Description of cohort:**

Project Viva is a longitudinal pre-birth cohort of mother-offspring pairs. We enrolled study participants between 1999 and 2002 from Atrius Harvard Vanguard Medical Associates, a multispecialty group practice in Eastern Massachusetts. Exclusion criteria included multiple gestation, inability to answer questions in English, gestational age ≥22 weeks at recruitment and plans to move away before delivery (28).

**Selected Sample for this analysis:**

For this analysis, as according to the analytic plan, we included only one child per mother and restricted the analysis to white participants only.

**Ethical approval:**

At the first study visit, which immediately followed the woman’s initial clinical prenatal visit, we obtained written informed consent, and the institutional review board of Harvard Pilgrim Health Care approved the project in line with ethical standards established by the Declaration of Helsinki. The IRB approval number for the Project Viva Epigenomics Data Repository is 671767. Mothers also provided genetic consent to use cord blood and mid-childhood blood for DNA methylation.

**EWAS analyst:**

Ruby Fore, Sheryl L. Rifas-Shiman

**Description of detail in FFQ:**

We provided a take-home self-administered questionnaire including a 166-item semi-quantitative FFQ assessing the woman’s diet during early pregnancy, defined as since her last menstrual period until FFQ completion date. We slightly modified the FFQ for use in pregnancy from the extensively validated FFQ used in the Nurses’ Health Study and other large cohort studies. The FFQ was validated by comparing FFQ responses to nutrient levels in pooled blood specimens among 204 participants (29). Maternal nutritional intake was registered at the first study visit, which immediately followed the woman’s initial clinical prenatal visit (mean 11.8 weeks; min-max 7.2-22.3 weeks). **Vegetables** included tomatoes, tomato juice, tomato sauce, salsa, string beans, broccoli, cabbage, cauliflower, brussel sprouts, raw carrots, cooked carrots, corn, peas or lima beans, mixed vegetables, dark squash, eggplant, yams, cooked spinach, raw spinach, kale, iceberg lettuce, leaf lettuce, celery, green pepper, garlic, and onion. **Legumes** included tofu, baked beans, and soymilk. We observed a skewed distribution for legumes consumption (with a large portion of the cohort never consuming them), thus all mothers who consumed 0 g/day were ranked in the first tertile. **Fruit/Nuts/Seeds** included raisins, dried fruit, prune, banana, cantaloupe, avocado, applesauce, apple or pear, cider, oranges, orange juice with calcium, regular orange juice, grapefruit, grapefruit juice, other juice, strawberry, blueberry, peach, peanuts, other nuts, and peanut butter. **Fish** included canned tuna, shrimp, dark fish, and other fish. **Cereals** included cold cereal, oatmeal, hot cereal, dark bread, brown rice, other grain, wheat germ, oat bran, other bran, white bread, bagels, muffins, white rice, pasta, tortilla, pancakes, graham cracker, and other crackers. **Meat** included bacon, chicken sandwich, other chicken, chicken no skin, beef hotdog, chicken hotdog, salami, processed meat, regular hamburger, lean hamburger, beef sandwich, pork, beef or lamb, beef liver, and chicken liver. **Dairy** included skim milk, 1or 2% milk, whole milk, cream, frozen yoghurt, ice cream, milkshake, flavored yoghurt, plain yoghurt, cottage or ricotta cheese, cream cheese, other cheese, and butter **Olive oil** included one question on olive oil. We derived 3-category olive oil as 0 servings/day = 0 (53% of participants) and among participants consuming any olive oil, <median = 1, >=median = 2. **Alcohol** was based on total alcohol intake. We derived the alcohol component as 5-25 grams/day = 2 vs. <5 or >25 g/d = 0, as specified in the analysis plan.

**Methylation normalization and QC steps taken in your cohort:**

*Cord blood and childhood blood samples:*

Cord blood samples collected at birth and childhood blood samples collected in mid-childhood were centrifuged within 24 hours of collection. Genomic DNA was extracted from nucleated cells using commercially available PureGene Kits (Fisher, Catalog Nos. A407-4, A416-4; Qiagen, Catalog Nos.158908, 158912, 158924), and frozen at -80 degrees C. Extracted DNA underwent bisulfite conversion using the Zymo EZ DNA Methylation kit (Zymo Research), and epigenome wide methylation was measured using the Illumina HM450K microarray. Data were preprocessed using the minfi package (15) in R (14). Failed samples, replicates, non-CpG probes, and probes on X and Y chromosomes were removed. Data were checked for gender mismatch using X and Y chromosomes. CpG sites with low detection p-values were identified and flagged. Raw methylation values were Noob adjusted (background and dye bias adjusted), and methylation values were normalized using a beta-mixture quantile normalization method.

**Covariates:**

**Maternal education** was self-reported at the initial prenatal visit and we dichotomized this covariate as at least a college degree v. less than a college degree. **Maternal smoking** during pregnancy was self-reported at first and at second trimester. Due to small number of women who were sustained smokers during pregnancy in our cohort, we ran the EWAS with the smoking covariate defined as ‘any smoking in pregnancy’ (versus non-smokers). **Newborn sex** was collected at the delivery interview. **Maternal BMI** was calculated based on self-reported maternal pre-pregnancy weight and height, registered at the initial prenatal visit. **Maternal total energy intake** was calculated in kcal/day by means of the self-administered FFQ. **Cell type composition** was estimated using the reference-based Houseman method (9) in the minfi Package (15) in R (14). This method estimates the relative proportions of white blood cell subtypes CD8T, CD4T, NK, Bcells, Monocytes, Granulocytes, nucleated red blood cells in cord blood (7). We used Combat (8) to adjust for **technical batch effect**, while protecting for the exposure of interest, for a total of 20 unique batches.

For the **look-up in childhood** (mean age 7.8y), we additionally adjusted for **childhood age** at DNA methylation measurement and **cell type composition** (CD8T, CD4T, NK, B cells, monocytes, granulocytes) was estimated using the Houseman method with the “Reinius” reference set (16,30).

**Funding:**

We received grants from the US National Institutes of Health (R01 HD034568, UH3 OD023286, R01 HL111108, R01 NR013945).

**Acknowledgements:**

We thank the participants and staff of Project Viva.

# References:

1. Boyd A, Golding J, Macleod J, Lawlor DA, Fraser A, Henderson J, Molloy L, Ness A, Ring S, Davey Smith G. Cohort Profile: the ’children of the 90s’--the index offspring of the Avon Longitudinal Study of Parents and Children. Int J Epidemiol. 2013;42:111–27.

2. Fraser A, Macdonald-Wallis C, Tilling K, Boyd A, Golding J, Davey Smith G, Henderson J, Macleod J, Molloy L, Ness A, et al. Cohort Profile: the Avon Longitudinal Study of Parents and Children: ALSPAC mothers cohort. Int J Epidemiol. 2013;42:97–110.

3. Relton CL, Gaunt T, McArdle W, Ho K, Duggirala A, Shihab H, Woodward G, Lyttleton O, Evans DM, Reik W, et al. Data resource profile: Accessible resource for integrated epigenomic studies (ARIES). Int J Epidemiol. 2015;44:1181–90.

4. Rogers I, Emmett P. Diet during pregnancy in a population of pregnant women in South West England. Eur J Clin Nutr. 1998;52:246–50.

5. Emmett PM, Jones LR, Golding J. Pregnancy diet and associated outcomes in the Avon Longitudinal Study of Parents and Children. Nutr Rev. 2015;73:154–74.

6. Min JL, Hemani G, Davey Smith G, Relton C, Suderman M. Meffil: efficient normalization and analysis of very large DNA methylation datasets. Hancock J, editor. Bioinformatics. 2018;

7. Gervin K, Salas LA, Bakulski KM, van Zelm MC, Koestler DC, Wiencke JK, Duijts L, Moll HA, Kelsey KT, Kobor MS, et al. Systematic evaluation and validation of reference and library selection methods for deconvolution of cord blood DNA methylation data. Clin Epigenetics. 2019;11:125.

8. Leek JT, Johnson WE, Parker HS, Fertig E, Jaffe AE, Storey JD, Zhang Y, Torres L. sva: Surrogate Variable Analysis. R package version 3.34.0. 2019;

9. Houseman EA, Accomando WP, Koestler DC, Christensen BC, Marsit CJ, Nelson HH, Wiencke JK, Kelsey KT. DNA methylation arrays as surrogate measures of cell mixture distribution. BMC Bioinformatics. 2012;13:1–16.

10. Kruithof CJ, Kooijman MN, van Duijn CM, Franco OH, de Jongste JC, Klaver CCW, Mackenbach JP, Moll HA, Raat H, Rings EHHM, et al. The Generation R Study: Biobank update 2015. Eur J Epidemiol. 2014;29:911–27.

11. Kooijman MN, Kruithof CJ, van Duijn CM, Duijts L, Franco OH, van IJzendoorn MH, de Jongste JC, Klaver CCW, van der Lugt A, Mackenbach JP, et al. The Generation R Study: design and cohort update 2017. Eur J Epidemiol. 2016;31:1243–64.

12. Klipstein-Grobusch K, Den Breeijen JH, Goldbohm RA, Geleijnse JM, Hofman A, Grobbee DE, Witteman JCM. Dietary assessment in the elderly: Validation of a semiquantitative food frequency questionnaire. Eur J Clin Nutr. 1998;

13. Lehne B, Drong AW, Loh M, Zhang W, Scott WR, Tan S-T, Afzal U, Scott J, Jarvelin M-R, Elliott P, et al. A coherent approach for analysis of the Illumina HumanMethylation450 BeadChip improves data quality and performance in epigenome-wide association studies. Genome Biol. 2015;16:37.

14. R Core Team. R Core Team (2014). R: A language and environment for statistical computing. R Found Stat Comput Vienna, Austria. 2014;R Foundation for Statistical Computing.

15. Aryee MJ, Jaffe AE, Corrada-Bravo H, Ladd-Acosta C, Feinberg AP, Hansen KD, Irizarry RA. Minfi: A flexible and comprehensive Bioconductor package for the analysis of Infinium DNA methylation microarrays. Bioinformatics. 2014;30:1363–9.

16. Reinius LE, Acevedo N, Joerink M, Pershagen G, Dahlén S-E, Greco D, Söderhäll C, Scheynius A, Kere J. Differential DNA Methylation in Purified Human Blood Cells: Implications for Cell Lineage and Studies on Disease Susceptibility. PLoS One. 2012;7:e41361.

17. Starling AP, Brinton JT, Glueck DH, Shapiro AL, Harrod CS, Lynch AM, Siega-Riz AM, Dabelea D. Associations of maternal BMI and gestational weight gain with neonatal adiposity in the Healthy Start study. Am J Clin Nutr. 2015;101:302–9.

18. Shapiro ALB, Schmiege SJ, Brinton JT, Glueck D, Crume TL, Friedman JE, Dabelea D. Testing the fuel-mediated hypothesis: maternal insulin resistance and glucose mediate the association between maternal and neonatal adiposity, the Healthy Start study. Diabetologia. 2015;58:937–41.

19. Moshfegh AJ, Rhodes DG, Baer DJ, Murayi T, Clemens JC, Rumpler W V., Paul DR, Sebastian RS, Kuczynski KJ, Ingwersen LA, et al. The US Department of Agriculture Automated Multiple-Pass Method reduces bias in the collection of energy intakes. Am J Clin Nutr. 2008;88:324–32.

20. Guxens M, Ballester F, Espada M, Fernández MF, Grimalt JO, Ibarluzea J, Olea N, Rebagliato M, Tardón A, Torrent M, et al. Cohort profile: The INMA-INfancia y Medio Ambiente-(environment and childhood) project. Int J Epidemiol. 2012;41:930–40.

21. Vioque J, Navarrete-Muñoz E-M, Gimenez-Monzó D, García-de-la-Hera M, Granado F, Young IS, Ramón R, Ballester F, Murcia M, Rebagliato M, et al. Reproducibility and validity of a food frequency questionnaire among pregnant women in a Mediterranean area. Nutr J. 2013;12:26.

22. Van Iterson M, Tobi EW, Slieker RC, Den Hollander W, Luijk R, Slagboom PE, Heijmans BT. MethylAid: Visual and interactive quality control of large Illumina 450k datasets. Bioinformatics. 2014;30:3435–7.

23. Fortin J-P, Fertig E, Hansen K. shinyMethyl: interactive quality control of Illumina 450k DNA methylation arrays in R. F1000Research. 2014;

24. Johnson WE, Li C, Rabinovic A. Adjusting batch effects in microarray expression data using empirical Bayes methods. Biostatistics. 2007;8:118–27.

25. USDA. National Nutrient Database for Standard Reference Release Legacy, release 23. Full Rep (All Nutr [Internet]. 2018; Available from: https://ndb.nal.usda.gov/ndb/

26. Imma Palma D, Farran A, Pilar Cervera S. Tablas de composición de alimentos por medidas caseras de consumo habitual en España. CESNID Madrid McGraw-Hill Interam. 2008;

27. Koestler DC, Jones MJ, Usset J, Christensen BC, Butler RA, Kobor MS, Wiencke JK, Kelsey KT. Improving cell mixture deconvolution by identifying optimal DNA methylation libraries (IDOL). BMC Bioinformatics. 2016;17:120.

28. Oken E, Baccarelli AA, Gold DR, Kleinman KP, Litonjua AA, Meo D De, Rich-Edwards JW, Rifas-Shiman SL, Sagiv S, Taveras EM, et al. Cohort profile: Project viva. Int J Epidemiol. 2015;44:37–48.

29. Fawzi WW, Rifas-Shiman SL, Rich-Edwards JW, Willett WC, Gillman MW. Calibration of a semi-quantitative food frequency questionnaire in early pregnancy. Ann Epidemiol. 2004;

30. Houseman EA, Accomando WP, Koestler DC, Christensen BC, Marsit CJ, Nelson HH, Wiencke JK, Kelsey KT. DNA methylation arrays as surrogate measures of cell mixture distribution. BMC Bioinformatics. 2012;13:1–16.
